# B cells secrete functional antigen-specific IgG antibodies on extracellular vesicles

**DOI:** 10.1038/s41598-024-67912-y

**Published:** 2024-07-23

**Authors:** Claudia Rival, Mahua Mandal, Kayla Cramton, Hui Qiao, Mohd Arish, Jie Sun, James V. McCann, Andrew C. Dudley, Michael D. Solga, Uta Erdbrügger, Loren D. Erickson

**Affiliations:** 1 Beirne Carter Center for Immunology Research, University of Virginia, PO Box 801386, Charlottesville, VA 22908 USA; 2 Department of Microbiology, Immunology, and Cancer Biology, University of Virginia, Charlottesville, VA 22908 USA; 3 Division of Infectious Diseases and International Health, Department of Medicine, University of Virginia, Charlottesville, VA 22908 USA; 4 Department of Cell Biology and Physiology, University of North Carolina at Chapel Hill, Chapel Hill, NC 27599 USA; 5 Emily Couric Cancer Center, University of Virginia, Charlottesville, VA 22908 USA; 6 Flow Cytometry Core, University of Virginia, Charlottesville, VA 22908 USA; 7 Division of Nephrology, Department of Medicine, University of Virginia, Charlottesville, VA 22908 USA

**Keywords:** Extracellular vesicles, B cells, Antibody production, Adaptive immunity, Adaptive immunity, Autoimmunity, Infection

## Abstract

B cells and the antibodies they produce are critical in host defense against pathogens and contribute to various immune-mediated diseases. B cells responding to activating signals in vitro release extracellular vesicles (EV) that carry surface antibodies, yet B cell production of EVs that express antibodies and their function in vivo is incompletely understood. Using transgenic mice expressing the Cre recombinase in B cells switching to IgG1 to induce expression of fusion proteins between emerald green fluorescent protein (emGFP) and the EV tetraspanin CD63 as a model, we identify emGFP expression in B cells responding to foreign antigen in vivo and characterize the emGFP^+^ EVs they release. Our data suggests that emGFP^+^ germinal center B cells undergoing immunoglobulin class switching to express IgG and their progeny memory B cells and plasma cells, also emGFP^+^, are sources of circulating antigen-specific IgG^+^ EVs. Furthermore, using a mouse model of influenza virus infection, we find that IgG^+^ EVs specific for the influenza hemagglutinin antigen protect against virus infection. In addition, crossing the B cell Cre driver EV reporter mice onto the *Nba2* lupus-prone strain revealed increased circulating emGFP^+^ EVs that expressed surface IgG against nuclear antigens linked to autoimmunity. These data identify EVs loaded with antibodies as a novel route for antibody secretion in B cells that contribute to adaptive immune responses, with important implications for different functions of IgG^+^ EVs in infection and autoimmunity.

## Introduction

Extracellular vesicles (EVs) are small 50–1000 nm membranous structures that are released by all cell types into the extracellular environment and are found in a variety of biological fluids^1^. These vesicles have emerged as integral mediators of intercellular communication and affect the functions of other cells through their surface proteins and bioactive cargo, such as proteins, lipids, and nucleic acids. Immune cell-derived EVs are associated with numerous physiological and pathological conditions, including infectious diseases and autoimmunity^2–4^. B cells are a heterogeneous population with functionally distinct subsets and contribute to adaptive immunity by the production of antibodies. There is clear evidence that B cells secrete EVs using cell lines and cultured primary cells. These foundational studies demonstrate the release of EVs from B cell lines and T cell- or mitogen-stimulated primary B cells which are enriched for MHC-II and components of the B cell receptor (BCR), including various tetraspanins and immunoglobulin^5–11^. Although EVs released by B cells have long been recognized, it has been studied primarily in the context of vesicle function in antigen presentation and mediating T cell responses. In comparison with B cell-derived EVs mediating cellular immunity, much less is known about B cells as a source of EVs in vivo and the contribution of these EVs to the secretion of biologically active antibodies and their potential relevance to the humoral immune response.

The production of IgG antibodies by B cells is an essential part of the adaptive immune response, providing specific and long-lasting protection after infections or vaccinations. The process begins when B cells recognize extracellular antigen by membrane-bound immunoglobulin on the B cell surface^12–14^. Upon encounter of T cell-dependent antigen, B cells are activated and form germinal centers (GC) where antigen-specific B cells undergo clonal expansion and differentiation into memory B cells and antibody-secreting plasma cells. Germinal centers also are the primary sites for antibody class switching, which leads to B cells changing the class of immunoglobulin they produce while retaining the specificity for the same antigen. Although IgG antibodies play a critical role in the dynamics of B cell responses after infection, the contribution of EVs derived from B cells expressing antigen-specific IgG antibodies is less well defined. Transgenic mice engineered to express Cre recombinase in B cells undergoing germline transcription of the constant part of the IgG1 chain (Cγ1) produce Cre recombinase in the majority of GC B cells in mice immunized with T cell-dependent antigen^15,16^. Here, using a novel emGFP reporter murine system that allows probing of CD63^+^ EVs derived from B cells in Cγ1-Cre mice, we rigorously demonstrate that B cells stimulated with polyclonal activating signals to promote switching to IgG1 secrete emGFP^+^ EVs that express surface IgG and CD63. In mice immunized with T cell-dependent antigen it was observed that GC B cells and their progeny memory B and plasma cells express emGFP and associated with circulating antigen-specific IgG^+^ emGFP^+^ EVs. Furthermore, we used two independent mouse models of disease to evaluate the functional capacity of antigen-specific IgG^+^ emGFP^+^ EVs in vivo. Influenza virus infection in mice can be neutralized by treatment with influenza A virus H1N1 A/PR8/34 (PR8) hemagglutinin-specific IgG^+^ EVs. By integrating the Cγ1-Cre driven EV reporter system with a spontaneous murine model of lupus we find that abnormal GC B cell responses are associated with the production of circulating EVs expressing surface pathogenic IgG autoantibodies.

## Results

### IgG1^+^ B cells of Cγ1^CD63-emGFP^ reporter mice express emGFP in vitro

**Figure 1:**
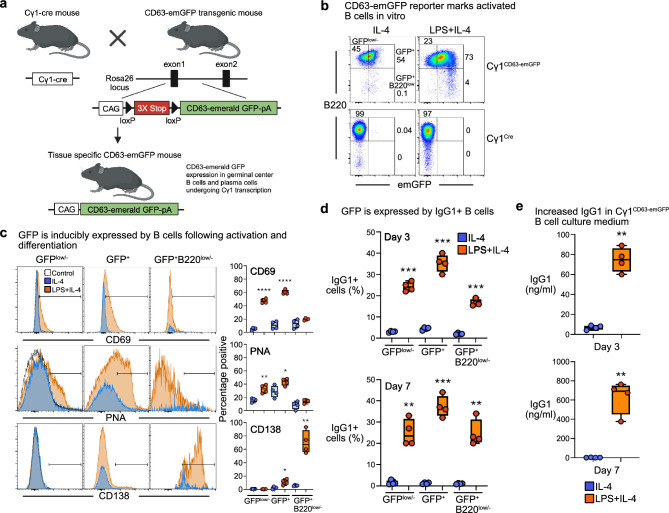
Activated Cγ1^CD63-emGFP^ B cells undergoing Cre-mediated recombination in vitro express emGFP. (**a**) Schematic for Cγ1^CD63-emGFP^ reporter strain design. Transgenic mice expressing Cre recombinase driven by transcription of the Ig γ1 constant region gene segment (Cγ1) was crossed with a silenced reporter mouse, resulting in CD63-emerald GFP expression driven by the CAG promoter. (**b**) Percentage of emGFP^+^ Cγ1^CD63-emGFP^ and Cγ1^Cre^ control B cells following stimulation with IL-4 or LPS + IL-4 for 3 days. Gates indicate GFP^low/-^ B cells, GFP^+^ B cells and GFP^+^B220^low/−^ B cells. (**c**) Percentage of cell surface expression of CD69, PNA and CD138 in GFP^low/−^, GFP^+^ and GFP^+^B220^low/−^ B cells at day 3, for the Cγ1^CD63-emGFP^ reporter mice in panel (**b**). Gates were set on negative control samples (dotted black histograms). (**d**) Frequencies of IgG1^+^ B cells in B cells gated on GFP expression at days 3 and 7, for the reporter mice in (**b**). (**e**) Concentration of IgG1 in the culture medium of B cells from the reporter mice in (**b**). All data are expressed as mean ± SEM. Results shown are representative of at least three independent experiments. *P* = **0.01 and ***0.001, with unpaired, two-tailed *t*-test.

To define EVs derived from mature activated B cells, we crossed CD63-emGFP^loxP/stop/loxP^ reporter mice^17^, a knock-in strain that takes advantage of the expression of fusion proteins between emerald green fluorescent protein (emGFP) and the tetraspanin CD63 in EVs, with Cγ1^Cre^ mice^15^ (Fig. 1a). We used this approach to distinguish EVs released by predominantly germinal center B cells undergoing germ-line Ig heavy constant gamma 1 (Cγ1) transcription and their progeny plasma cells and memory B cells, from EVs of immature and naïve B cells for in vivo analyses. To test whether emGFP is inducibly expressed by Cre recombinase in B cells, we initially used polyclonal B cell stimuli to drive IgG1 antibody class switching in vitro. Splenic B cells from Cγ1^CD63-emGFP^ mice and Cγ1^Cre^ littermate controls were stimulated with bacterial lipopolysaccharide (LPS) to mimic T cell-independent B cell activation and the addition of interleukin 4 (IL-4) to induce class switching to IgG1^18^, and assessed 3 days later for emGFP expression by flow cytometry. Unstimulated controls consisted of splenic B cells from Cγ1^CD63-emGFP^ mice and Cγ1^Cre^ mice cultured with IL-4 alone. A fraction (~ 75%) of Cγ1^CD63-emGFP^ B cells expressed emGFP in the presence of LPS + IL-4 compared to B cells from Cγ1^Cre^ controls (Fig. 1b). emGFP expression was induced in Cγ1^CD63-emGFP^ B cells (~ 54%) stimulated with IL-4 though not to the degree measured in LPS + IL-4 cultures. This suggests that IL-4, in the absence of stimulation through the BCR and/or coreceptors, promotes a portion of B cells to undergo transcription from the Cγ1 promoter, which has previously been observed^15,18,19^. As expected, IL-4 alone or with LPS did not induce emGFP expression in control Cγ1^Cre^ B cells (Fig. 1b), and no emGFP expression was detected in splenic non-B cells (Supplementary Fig. S1a). Upon LPS + IL-4 stimulation, CD69 and lectins that bound peanut agglutinin (PNA), which are hallmarks of activation, were induced on a portion of emGFP^low^ B cells and even a larger portion of emGFP^+^ B cells (Fig. 1c). It also promoted the differentiation of B220^low/-^CD138^+^ plasma cells that expressed emGFP (Fig. 1c). In contrast, IL-4 alone induced less activation and plasma cell differentiation of B cells as measured by these markers. We next tested whether B cells expressing membrane IgG1 had variable levels of emGFP expression based on these activation and differentiation phenotypes. Quantitative analysis of membrane IgG1 expression on activated (emGFP^+^) and differentiated (emGFP^+^B220^low/−^) B cells following stimulation with LPS + IL-4 confirmed these were IgG1-switched cells starting at day 3 through day 7 of culture (Fig. 1d). As expected, given the increased frequencies of activated emGFP^+^ B cells, the frequencies of these cells that expressed membrane IgG1 were higher compared to emGFP^+^B220^low/-^ plasma cells that are terminally differentiated and ultimately secrete IgG1. Some membrane IgG1^+^ B cells were emGFP^low^, which suggested that these were activated B cells that recently expressed IgG1 germline and switch transcripts. Together, of heterogeneous LPS-responding B cells, activated and differentiated membrane IgG1^+^ B cells expressing varying levels of emGFP represent ~ 80% of cultured B cells. Lastly, the expression of emGFP in IgG1^+^ activated B cells and plasma cells from LPS + IL-4 cultures coincided with the presence of soluble IgG1 in the cell culture medium at day 3 and substantially higher levels at day 7 of culture (Fig. 1e). As expected, control Cγ1^Cre^ B cells stimulated with LPS + IL-4 also produced soluble IgG1 in the cell culture medium (Supplementary Fig. S1b). Consistent with what was observed for LPS + IL-4, Cγ1^CD63-emGFP^ B cells stimulated with anti-CD40 mAb and IL-4 to mimic T cell-dependent B cell activation that promotes IgG1 switching^20^ also induced emGFP expression in B cells from Cγ1^CD63-emGFP^ but not control mice (Supplementary Fig. S1c). Collectively, these data suggest that transcription from the Cγ1 locus induces efficient Cre-mediated recombination in vitro in B cells to express emGFP using the CD63-emGFP^loxP/stop/loxP^ reporter allele and that the transgene does not affect B cell activation and differentiation.

### IgG1^+^ B cells of Cγ1^CD63-emGFP^ reporter mice express emGFP in vivo

**Figure 2:**
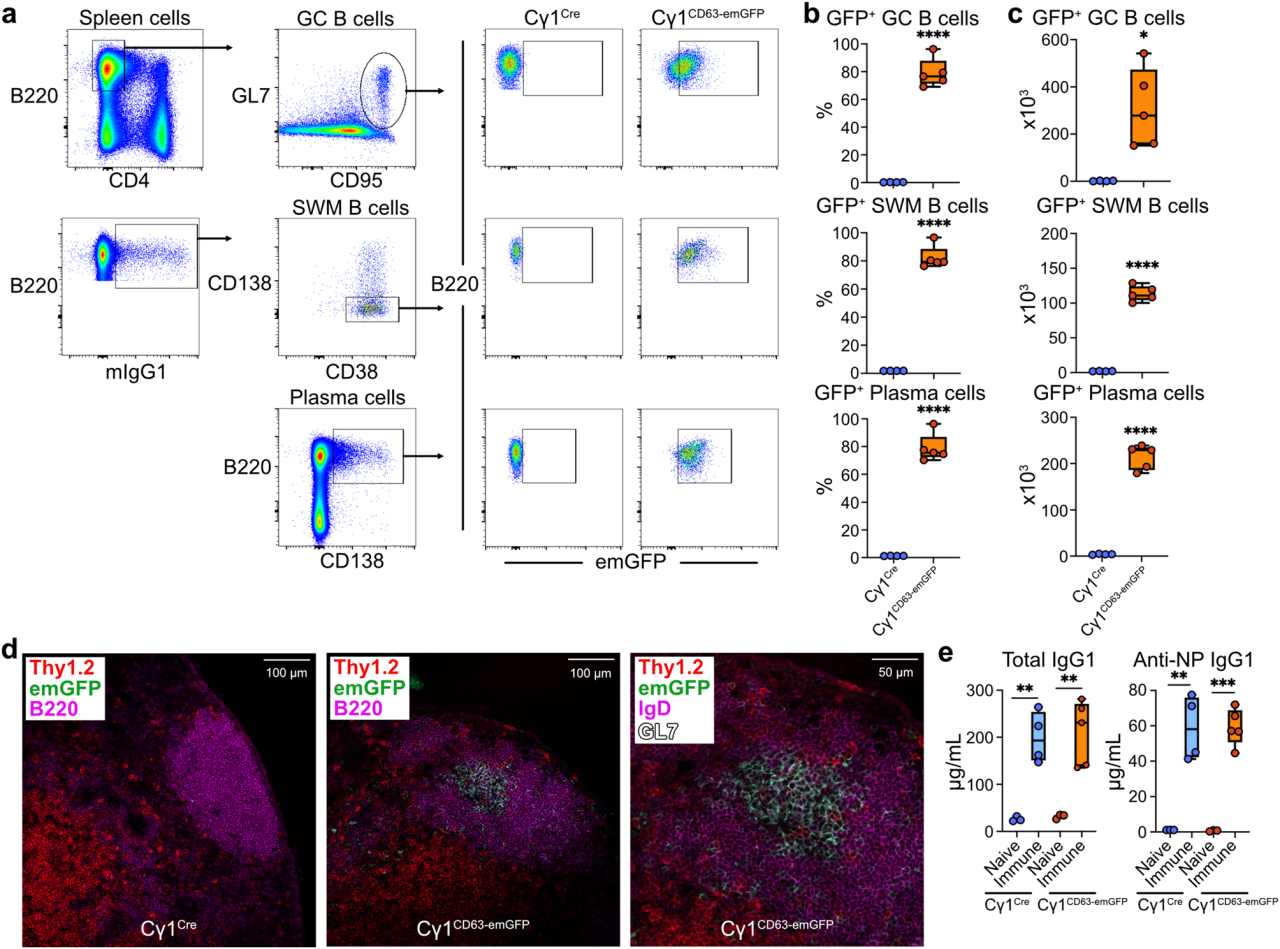
In vivo generation of Cγ1^CD63-emGFP^ GC B cells and progeny IgG1^+^ memory B cells and plasma cells express emGFP after immunization with NP-KLH. (**a**) Flow cytometry gating strategy of GC B cells, IgG1^+^ SWM B cells, and plasma cells that express emGFP in the spleens of Cγ1^Cre^ and Cγ1^CD63-emGFP^ mice immunized with NP-KLH for 14 days. (**b**) Percentages of emGFP^+^ cells within each gated B cell subset are shown. (**c**) Numbers of emGFP^+^ B cells in spleens for the reporter mice shown in panel (**b**). (**d**) Immunofluorescence confocal microscopy of emGFP^+^ B cells in the lymph nodes of mice immunized with NP-KLH for 14 days. (**e**) Serum total and NP-specific IgG1 antibody levels in naïve Cγ1^Cre^ and Cγ1^CD63-emGFP^ mice and after immunization with NP-KLH for 14 days. Data are expressed as mean ± SEM. Results shown in panels (**a**–**c**) and (**e**) are representative of three independent experiments and in panel d from > 20 images of two independent experiments. *P* = *0.05, **0.01, ***0.001 and ****0.0001, with unpaired, two-tailed *t*-test.

To determine the efficiency of Cre-mediated recombination in Cγ1^CD63-emGFP^ reporter mice in vivo, mice were immunized with the T cell-dependent hapten-carrier NP-KLH in alum and the spleens were analyzed 14 days later by flow cytometry. Consistent with prior findings using Cγ1^Cre^ mice bred to the Rosa26-EYFP strain^15^, in spleens of immunized Cγ1^CD63-emGFP^ mice, most GC B cells expressed emGFP (Fig. 2a–c). Further, switched memory B cells (B220^+^IgG1^+^CD138^-^CD38^+^) and immature plasma cells (B220^+^CD138^+^) expressed emGFP. As expected, emGFP expression was not detected in GC B cells or progeny switched memory B cells and plasma cells from immunized Cγ1^Cre^ littermate control mice (Fig. 2a–c), even though they generated these B cell subsets at frequencies similar to immunized Cγ1^CD63-emGFP^ mice (Supplementary Fig. S2a). No emGFP expression was detected in B cells from naïve Cγ1^CD63-emGFP^ mice, or in T cells and marginally in non-B cells of immunized Cγ1^CD63-emGFP^ mice (Supplementary Fig. S2b,c), supporting the specificity of the Cre-mediated recombination to antigen-experienced B cells in vivo. Immunofluorescence staining of frozen lymph node sections from immunized Cγ1^CD63-emGFP^ mice showed increased frequencies of emGFP^+^ cells within B220^+^ and IgD^+^ B cell follicles that were brightly stained with the GC marker GL7 (Fig. 2d). No emGFP expression in B cells was detected in littermate control mice. Measurement of total and NP-specific IgG1 antibody levels by ELISA revealed higher amounts in sera from immune mice compared to naïve controls regardless of genotype (Fig. 2e). Thus, in Cγ1^CD63-emGFP^ mice immunized with a T cell-dependent antigen, Cre-mediated recombination occurs largely within the GC B cell population and in IgG1^+^ memory B cells and plasma cells. Given these findings, our focus shifted to the examination of EVs released from emGFP^+^ B cells.

### EVs derived from B cells of Cγ1^CD63-emGFP^ reporter mice express emGFP and IgG

**Figure 3:**
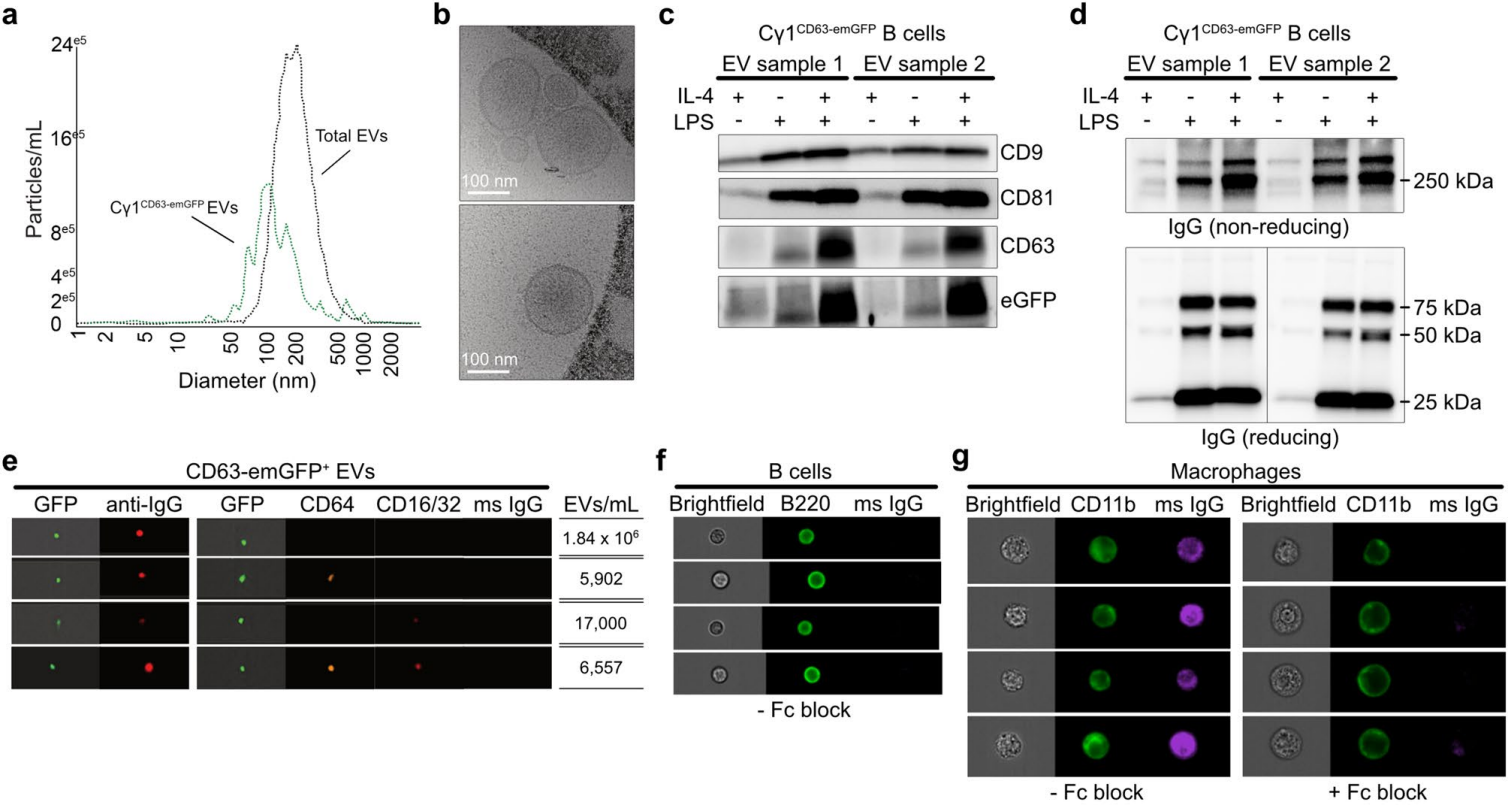
Activated Cγ1^CD63-emGFP^ B cells undergoing Cre-mediated recombination in vitro produce EVs that express emGFP, tetraspanins, and IgG. (**a**) Nanoparticle tracking analysis of total and GFP^+^ EVs isolated from cell culture medium from Cγ1^CD63-emGFP^ B cells stimulated with LPS + IL-4 for 7 days. (**b**) EVs were visualized by cryoelectron microscopy. Images are representative of three independent EV preparations. (**c**, **d**) Lysates from EVs isolated from cell culture medium from Cγ1^CD63-emGFP^ B cells stimulated with IL-4, LPS, and LPS + IL-4 for 7 days were probed by Western blot for the presence of CD9, CD81, CD63, GFP, and IgG under non-reducing conditions, and IgG under reducing conditions (10 μg/lane). Under reducing conditions, IgG bands from EV samples 1 and 2 are juxtaposed from the same blot as indicated by vertical line, from full length blots shown in Supplementary Fig. S6b. Results show two EV preparations and are representative of at least six independent experiments. (**e**) ImageStream analysis of individual emGFP^+^ EVs isolated from the cell culture medium of Cγ1^CD63-emGFP^ B cells stimulated with LPS + IL-4 and stained for surface IgG or CD16/32 (Alexa Fluor 647), CD64 (PE), and binding of exogenous mouse IgG (DyLight 405). (**f**) ImageStream analysis of peritoneal B cells stained for surface B220 (FITC) and binding of exogenous mouse IgG. (**g**) ImageStream analysis of peritoneal macrophages stained for surface CD11b (FITC) and binding of exogenous mouse IgG in the absence (left panel) or presence of 2.4G2 Fc block (right panel). The results shown are representative of three independent experiments.

Heterogeneous expression of various BCR components is found in EVs produced from B cell lymphoma cell lines and in murine primary B cells activated in vitro^8,9,21^. Thus, to assess EVs released from Cγ1^CD63-emGFP^ B cells that have undergone Cre-mediated recombination, we isolated EVs from the cell culture medium of B cells stimulated with LPS and IL-4 together, in which a subset of the cells was determined to express emGFP and IgG in Fig. 1, using differential centrifugation. Measurement of the size distribution and presence of emGFP of the B cell-derived EVs by nanoparticle tracking analysis indicated that the majority of total EVs regardless of emGFP expression were between 50 and 500 nm with a median diameter of ~ 150 nm (Fig. 3a), suggesting that they comprise a heterogeneous group of EVs including exosomes and ectosomes. Further analysis of EVs that express emGFP indicated a smaller size distribution with a median diameter of ~ 120 nm. We also visualized these isolated EVs by microscopy as another indicator for the size and morphology of vesicles. Cryo-electron microscopy of EVs showed vesicular structures with bilayer membranes that were mainly in the size range of 100–200 nm (Fig. 3b). To further validate the isolated EVs, we performed western blot analysis for the presence of the canonical EV markers CD9, CD63, and CD81. Lysates were prepared from the same amount of isolated EVs from the cell culture medium of B cells stimulated for 7 days in vitro with IL-4 and LPS. All three EV markers were highly expressed in the EV lysates of B cells stimulated with LPS + IL-4 (Fig. 3c; Supplementary Fig. S3). Importantly, emGFP expression was strongly detected in EVs from B cells stimulated with LPS + IL-4 and showed a migrating band at ~ 50 kDa corresponding to the expected molecular weight of ~ 50–60 kDa of CD63 (Supplementary Fig. S3), indicating the presence of the CD63:emGFP fusion protein within EVs and is consistent with the original report using the CD63-emGFP^loxP/stop/loxP^ reporter mice^17^. The slightly higher molecular bands in the lysate are likely glycosylated products since CD63 is known to be post-translationally modified^22^. With the anti-GFP antibody, the lower molecular bands in the lysate are likely a GFP cleavage product based on a previous report with cells transfected with the emGFP vector alone^17^. CD9, but not CD81, CD63 or emGFP was reliably detected in EVs from B cells cultured with IL-4 thus confirming that LPS stimulation was needed to promote Cre-mediated class switch recombination in B cells for emGFP labeling and increase secretion of CD63^+^ EVs (Fig. 3c; Supplementary Fig. S4). Given that a subset of B cells of Cγ1^Cre^ mice can undergo Cre-mediated class switch recombination to other IgG isotypes^15,23^, we wondered whether the expression of EV-associated markers and emGFP was detected in EVs isolated from the cell culture medium of Cγ1^CD63-emGFP^ B cells stimulated with only LPS that induces switching to IgG3 and IgG2b^15,24,25^. CD9 and CD81 were detected in EVs from B cells cultured with LPS similar to levels detected in EVs from B cells cultured with LPS + IL-4 (Fig. 3c). In contrast, CD63 and emGFP detection was substantially lower in EVs from B cells cultured with LPS compared to LPS + IL-4 cultures, indicating that class switching to IgG3 and IgG2b in B cells leads to inefficient Cre-mediated recombination and low levels of CD63 and emGFP within EVs. Ponceau staining revealed similar protein profiles for all three culture conditions thus confirming the equal loading of EV lysates (Supplementary Fig. S5). Based on these findings we conclude that EVs released from B cells stimulated with IL-4 alone and in the presence of LPS show the typical characteristics of EVs and comprise a heterogeneous group of EVs.

To test whether EVs enriched for CD63 and emGFP express IgG, we probed lysates from EV samples under non-reducing and mild reducing conditions to evaluate the characteristics of IgG. IgG was highly abundant in EV samples isolated from B cells stimulated with LPS and LPS + IL-4 and predominantly migrated at a high molecular weight (> 250 kDa) in the presence of SDS under non-reducing conditions (Fig. 3d; Supplementary Figs. S6 and S7). Slight bands at 25, 50, and 150 kDa were also detected, suggesting that inter-chain disulfide bonds of the intact IgG are a result of reduction under native conditions. As expected, IgG was present at low levels in EVs isolated from the cell culture medium of B cells stimulated with IL-4 and is consistent with low soluble IgG measured in the same medium (Fig. 1e). Under reducing conditions, the high molecular weight IgG species were dissociated, indicating that the molecules were covalently linked by disulfide bonds. IgG light and heavy chains were detected in EV samples from B cells stimulated with LPS and LPS + IL-4 at the expected molecular weights of 25 kDa and 50 kDa, respectively. The 75 kDa band found in the lysates in reducing conditions only likely corresponds to one half of an IgG antibody comprised of a heavy chain and a light chain, as previously reported^26,27^. The high molecular weights of IgG from lysates of EVs isolated from the cell culture medium were also found in cultured B cells, which were dissociated to IgG light and heavy chains of the same molecular weights under reducing conditions (Supplementary Fig. S7). As expected, IgG in lysates isolated from B cells cultured with LPS + IL-4 also showed a migrating band at 150 kDa corresponding to monomeric IgG (Supplementary Fig. S7a). Together, these data indicate that EVs derived from B cells of Cγ1^CD63-emGFP^ reporter mice contain IgG and suggest IgG aggregate formation based on the > 250 kDa high molecular weight IgG species. Lastly, EVs isolated from the cell culture medium of Cγ1^CD63-emGFP^ B cells stimulated with LPS + IL-4 were stained for IgG and analyzed by ImageStream^®^ flow cytometry. Data showed that emGFP^+^ EVs also expressed surface IgG (Fig. 3e). As controls, buffer only or mAb cocktail only did not show any emGFP^+^ and/or IgG^+^ vesicles (Supplementary Fig. S8). IgG Fc gamma binding receptors CD64 and CD16/32 are expressed in murine B-lineage progenitor cells but not in mature B cells^28,29^. We therefore analyzed EVs for the expression of these molecules. As expected, the majority of emGFP^+^ EVs did not express CD64 and CD16/32 (Fig. 3e). To test whether emGFP^+^ EVs bind exogenous IgG, EVs were incubated with fluorescently conjugated mouse monomeric IgG. emGFP^+^ EVs showed undetectable expression of exogenous IgG (Fig. 3e; Supplementary Fig. S9) and is consistent with the lack of IgG binding by mature B220^+^ B cells (Fig. 3f). Analysis of mouse peritoneal macrophages, which express Fc gamma receptors^30^, confirmed the binding capacity of exogenous IgG on ~ 98.6% of CD11b^+^ macrophages (Fig. 3g; left panel). Importantly, pre-treatment of cells with the 2.4G2 monoclonal antibody to prevent binding of the Fc portion of IgG to the Fc receptors prior to incubation with exogenous mouse IgG showed undetectable levels of fluorescent IgG on CD11b^+^ macrophages (0.1%), indicating the binding of exogenous IgG was mediated via its Fc domain (Fig. 3g; right panel). Additionally, analysis of emGFP^-^ EVs demonstrated the binding capacity of exogenous IgG on EVs likely derived from immature B cells and/or non-B cells (Supplementary Fig. S9). These data support the notion that Cre-mediated recombination of Cγ1^CD63-emGFP^ B cells leads to the secretion of EVs that contain emGFP and IgG, including surface IgG expression that is not through CD64 and CD16/32 binding.

### emGFP-expressing EVs display antigen-specific IgG on the surface and bind antigen

**Figure 4:**
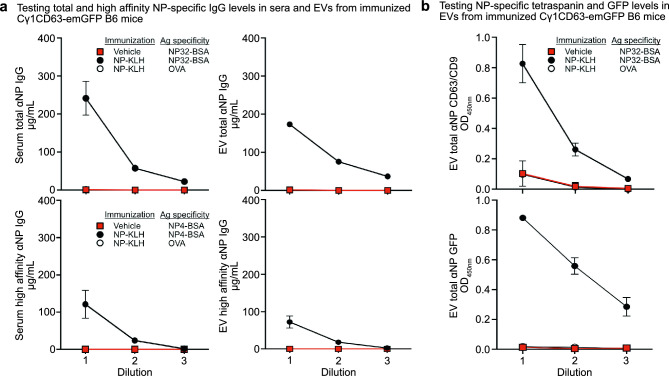
Circulating EVs produced from Cγ1^CD63-emGFP^ B cells bind specific antigen and express GFP and EV tetraspanin markers after immunization with NP-KLH. (**a**) ELISA measurements of NP-specific total (NP32-BSA) and high-affinity (NP4-BSA) IgG antibody levels in whole serum (left two panels) and EVs isolated from serum (right two panels) from Cγ1^CD63-emGFP^ mice unimmunized or immunized with NP-KLH for 14 days. Sample dilutions shown are 1:1000–1:30,000 for serum and 3.3 × 10^7^–3.7 × 10^6^ particles/ml for EVs. (**b**) ELISA measurements for the presence of both CD63 and CD9 and GFP from NP-specific EVs, for the reporter mice in panel (**a**). Data are expressed as mean ± SEM. The results shown are representative of three independent experiments.

Prompted by our in vitro data, that emGFP^+^ EVs expressed surface IgG, we therefore analyzed EVs isolated from sera of Cγ1^CD63-emGFP^ mice after immunization with NP-KLH for the presence of NP-specific IgG expressing EVs by ELISA. Two different molar ratios of NP per BSA carrier protein were used to determine binding of total NP-specific (NP32) and high-affinity NP-specific (NP4) IgG antibodies. First, we confirmed that total and high-affinity NP-specific IgG levels were detected in sera from immunized mice (Fig. 4a). Low to undetectable levels of NP-specific IgG were found in sera from naïve (vehicle) control mice or when the irrelevant OVA carrier protein was used as capture, further confirming NP specificity of the IgG response in immunized animals. Similar to our observations in sera, EVs isolated from sera of immunized mice showed increased levels of total and high-affinity NP binding IgG antibodies compared to EVs from naïve mice and when using OVA as capture antigen. Equal numbers of EVs (3.3 × 10^7^ to 3.7 × 10^6^ particles/ml) based on nanoparticle tracking analysis were used at serial dilutions in these assays to ensure that any differences in the results were not due to variations in the number of EVs used. We further found that EVs from immunized mice bound to NP also co-expressed CD9 and CD63 as well as emGFP (Fig. 4b). Thus, circulating EVs released from Cγ1^CD63-emGFP^ expressing B cells display these EV markers along with IgG on the surface that can bind antigen. These results also suggest that high-affinity NP-binding EVs are produced by activated emGFP^+^ B cells that have undergone affinity maturation.

### EVs expressing HA-specific IgG promote neutralization in influenza infected mice

**Figure 5:**
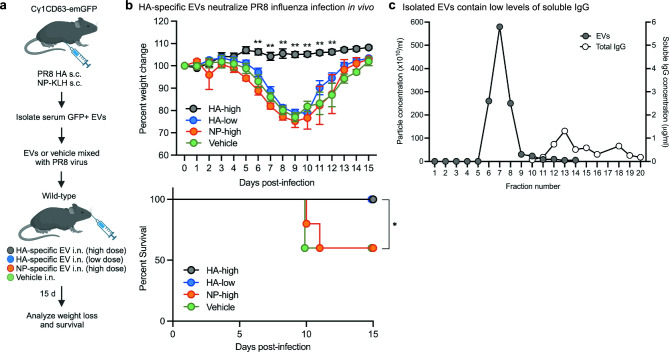
HA-specific EVs neutralize influenza infection. (**a**) Experimental strategy for testing HA-specific EVs in influenza infection in vivo. (**b**) Four groups of mice (*n* = 6 mice per group) were intranasally infected with PR8 virus only (Vehicle), PR8 virus mixed with high dose of EVs isolated from serum from Cγ1^CD63-emGFP^ mice immunized with influenza A hemagglutinin (HA) protein for 21 days (HA-high), PR8 virus mixed with low dose of EVs from the same mice immunized with HA (HA-low), or PR8 virus mixed with high dose of EVs isolated from serum from Cγ1^CD63-emGFP^ mice immunized with NP-KLH for 21 days. Host survival and body weight change following PR8 challenge were monitored. Data are expressed as mean ± SEM. The results shown are representative of two independent experiments. *P* = **0.001 between the HA-high and HA-low body weight change, calculated by unpaired, two-tailed *t*-test. *P* = * < 0.05 for survival in HA-high and HA-low groups as compared with NP-high and Vehicle groups, calculated by log-rank test. (**c**) Analysis of EV samples from serum from Cγ1^CD63-emGFP^ mice immunized with HA protein by size exclusion chromatography collected in 0.5 ml fractions. The number of particles per fraction was measured by nanoparticle tracking analysis and the amount of soluble IgG per fraction was measured by ELISA. Results shown are representative of two independent experiments.

IgG antibodies are known to be important in antiviral immune responses during influenza infection^31–33^. Most influenza vaccines mediate strain-specific protection by generating neutralizing antibody responses to hemagglutinin (HA), one of the surface glycoproteins of the virus. We sought to determine whether EVs carrying influenza-specific IgG antibodies have neutralization activity against an influenza virus challenge. We used a mouse model of influenza A virus H1N1 A/PR8/34 (PR8) infection, in which viral clearance occurs within 10 days post-infection^34–36^. We immunized Cγ1^CD63-emGFP^ reporter mice with PR8-specific recombinant HA protein, and as controls, with NP-KLH, and isolated EVs from serum pools from immunized mice (Fig. 5a). Analysis of HA- and NP-specific IgG levels in pooled sera and EVs isolated from pooled sera from immunized mice showed induced levels (Supplementary Fig. S10a,b). Antigen-specific EVs were further confirmed to express the EV markers CD63, CD81, CD9, and emGFP (Supplementary Fig. S10c and data not shown). EVs from HA- and NP-immunized mice at either a high (3 × 10^12^) or a thousand times lower (3 × 10^9^) dose were mixed with influenza PR8 virus and administered intranasally to naïve wild-type mice. The group of mice that were infected with a low dose of HA-specific EV and PR8 virus mixture showed severe body weight loss, but all survived and recovered by day 15 after infection (Fig. 5b). Mice infected with a high dose of HA-specific EV and PR8 virus mixture did not show body weight loss and were fully protected against a lethal infection. In contrast, mice that were infected with a high dose of NP-specific EV and PR8 virus mixture or PR8 virus alone showed severe body weight loss, and 40% of mice in each group succumbed by day 10 after infection.

To determine whether HA-specific EV samples contained contaminating levels of soluble IgG that might contribute to the neutralization of PR8 virus, EVs isolated by differential ultracentrifugation from pooled sera of HA-immunized mice were subsequently separated from soluble proteins by size-exclusion chromatography^37^. The total IgG concentration in each fraction containing soluble proteins (fractions 12–20) was measured by ELISA, and the number of particles per fraction was measured by nanoparticle tracking analysis. EVs eluted first and were found in fractions 6–9, while 1.3 μg/ml or less of soluble IgG was detected in fractions 12 onwards, totaling ~ 2.5 μg in fractions 12–20 combined (Fig. 5c). This concentration of soluble IgG present in EV samples is substantially low compared with mouse serum that typically contains greater than 5 mg/ml of IgG (Ref.^38^ and data not shown), indicating that most soluble IgG was removed during the process of isolating EVs by differential centrifugation. Further, the amount of soluble IgG in the high or low dose of HA-specific EVs mixed with PR8 virus would be minimal given the small volume of EVs (20 μl) used for each host. These results support that the main mechanism of protection against virus infection using this experimental approach is through neutralizing IgG antibodies against the influenza HA protein expressed by EVs.

### emGFP^+^ EVs expressing antinuclear IgG antibodies are produced from spontaneous germinal center B cell responses in lupus-prone mice

**Figure 6:**
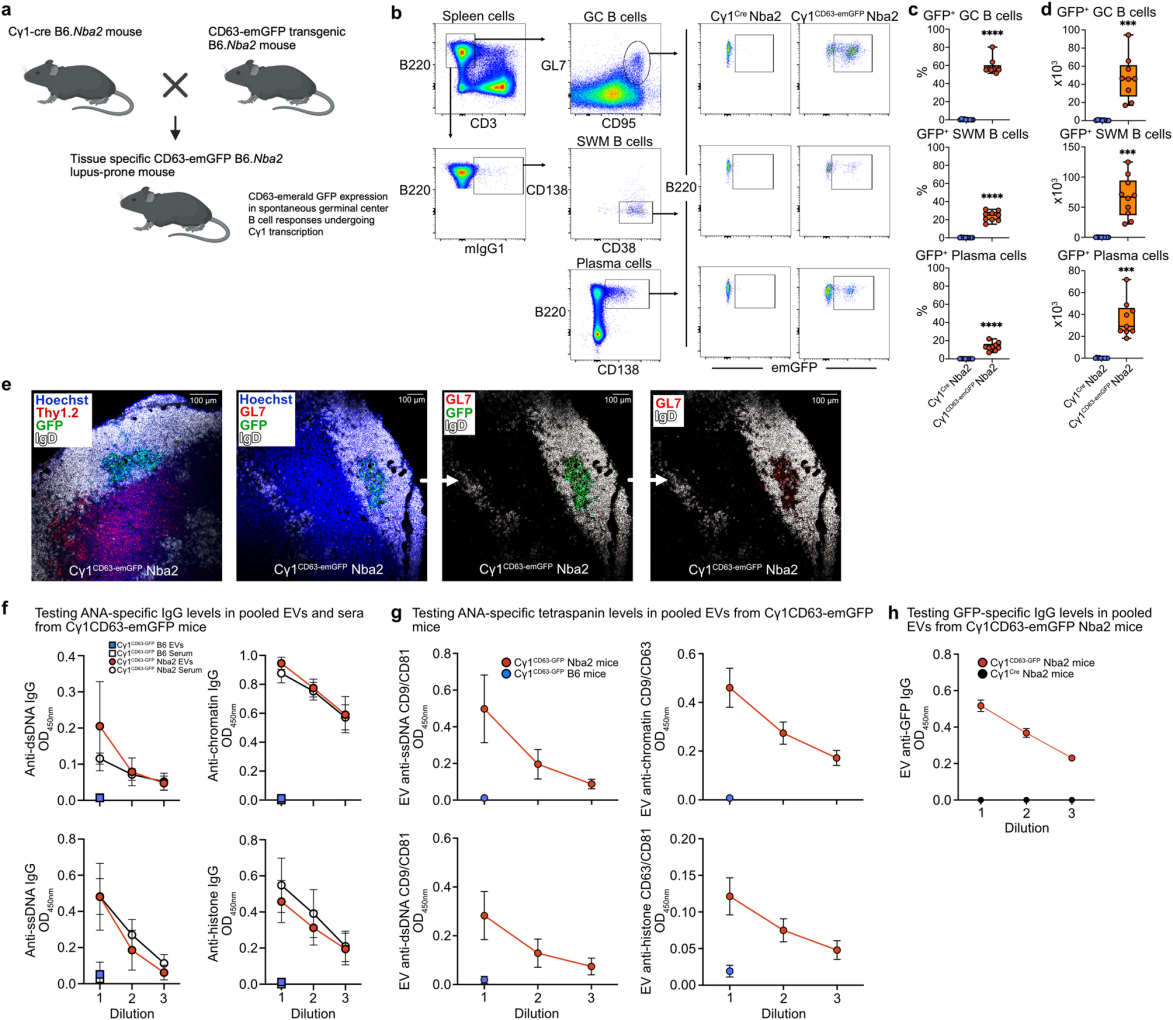
EVs derived from spontaneous germinal center B cell responses in Cγ1^CD63-emGFP^ *Nba2* lupus-prone mice express surface IgG with self-reactivity. (**a**) Schematic for generating the Cγ1^CD63-emGFP^ reporter strain on the *Nba2* lupus-prone congenic background. (**b**) Flow cytometry gating strategy of GC B cells, IgG1^+^ SWM B cells, and plasma cells that express emGFP in the spleens of 7-mo-old Cγ1^Cre^ *Nba2* and Cγ1^CD63-emGFP^ *Nba2* mice. (**c**) Percentages of emGFP^+^ cells within each gated B cell subset are shown (*n* = 9 mice per group). (**d**) Numbers of emGFP^+^ B cells in spleens for the reporter mice shown in panel (**c**). (**e**) Immunofluorescence confocal microscopy of emGFP^+^ B cells in the spleens of 7-mo-old Cγ1^CD63-emGFP^ *Nba2* mice. (**f**) ELISA measurements of antinuclear IgG antibody levels in whole serum and EVs isolated from serum from Cγ1^CD63-emGFP^ *Nba2* mice and Cγ1^CD63-emGFP^ B6 controls, for the reporter mice in panel (**c**). Sample dilutions shown are 1:500–1:3000 for serum and 6.6 × 10^8^–7.4 × 10^7^ particles/ml for EVs. (**g**, **h**) ELISA measurements for the presence of antinuclear binding EVs that express either CD9 and CD81, CD63 and CD9, or CD63 and CD81, and emGFP from EVs, for the reporter mice in panel (**c**). Data are expressed as mean ± SEM. The results shown in panels (**b**–**d**) and (**f**–**h**) are representative of three independent experiments, and in panel e from two independent experiments. *P* = ***0.001 and ****0.0001, with unpaired, two-tailed *t*-test.

Autoantibodies are critical in the pathogenesis of antibody-mediated autoimmune disorders. B6.*Nba2* congenic mice express the autoimmune susceptibility locus *Nba2* that is associated with loss of immune tolerance on the C57BL/6 background and spontaneously develop GCs and serum anti-nuclear IgG autoantibodies with the highest levels observed in 7-mo-old females^39–43^. To test whether spontaneously activated B cells release EVs that express IgG autoantibodies, we crossed CD63-emGFP^loxP/stop/loxP^ reporter B6.*Nba2* mice with Cγ1^Cre^ B6.*Nba2* mice (Fig. 6a). Analysis of spleens from 7-mo-old female Cγ1^CD63-emGFP^ *Nba2* reporter mice showed that both the percentages and the numbers of emGFP^+^ B cells significantly increase in a subset of GC B cells, switched memory B cells, and plasma cells compared to Cγ1^Cre^ *Nba2* controls (Fig. 6b–d). The total numbers of GC B cells, switched memory, and plasma cells were similar between Cγ1^Cre^ *Nba2* mice and Cγ1^CD63-emGFP^ *Nba2* mice (Supplementary Fig. S11a), demonstrating that the transgene does not cause any dominant effects on spontaneous B cell differentiation. No emGFP expression was detected in T cells from Cγ1^CD63-emGFP^ *Nba2* mice (Supplementary Fig. S11b), supporting the specificity of the Cre-mediated recombination to B cells in vivo in this model. Immunofluorescence staining of frozen spleen sections from Cγ1^CD63-emGFP^ *Nba2* mice showed GFP^+^ IgD^-^ cells within IgD^+^ B cell follicles bordering Thy1.2^+^ T cells that positively stained for the GC marker GL7 (Fig. 6e). emGFP^+^ B cells were mostly found in germinal centers with few to virtually none detected in T cell areas in the spleen. Measurement of antinuclear IgG autoantibodies by ELISA confirmed elevated levels in sera from Cγ1^CD63-emGFP^ *Nba2* mice compared to non-autoimmune Cγ1^CD63-emGFP^ B6 mice (Fig. 6f). EVs isolated from sera of the same animals showed a similar pattern with increased levels of antinuclear antigen binding IgG. Further, EVs from Cγ1^CD63-emGFP^ *Nba2* mice that bound to nuclear antigens also expressed EV tetraspanins and emGFP compared to control animals (Fig. 6g,h). Thus, in Cγ1^CD63-emGFP^ *Nba2* mice, Cre-mediated recombination for emGFP expression occurs spontaneously in a subset of GC B cells, plasma cells, and switched memory B cells. These results also provide support that emGFP^+^ autoreactive B cells produce EVs that express surface IgG and bind nuclear antigens. Thus, both self-antigens in the Cγ1^CD63-emGFP^ *Nba2* lupus-prone model and foreign antigen in the Cγ1^CD63-emGFP^ B6 model immunized with NP-KLH show emGFP expression in GC B cells, SWM B cells, and plasma cells. The frequencies of these B cell subsets between these two models are explained by the differences in genetic susceptibility on chromosome 1, the specific antigens and adjuvant used, and the kinetics of the immune response.

## Discussion

Here we have used Cγ1^CD63-emGFP^ reporter mice to study the in vitro and in vivo release of EVs from activated primary B cells undergoing IgG1 switching in response to T cell-independent and -dependent stimuli, during infection with influenza virus, and in spontaneous murine lupus. These Cγ1^CD63-emGFP^ reporter mice allowed the direct identification of B cells undergoing transcription at the Cγ1 locus by emGFP expression, which we observed in vitro is induced in activated B cells responding to T cell-independent and T cell-dependent stimuli that promote IgG1 class switching. We also reveal GC B cells as major participants undergoing Cre-mediated recombination after immunization with the model T cell-dependent antigen NP-KLH. This finding is consistent with previous reports identifying the germinal center as the major site for class switch recombination, including the switch to IgG1^15,44^. Notably, as progeny of GC B cells that switched to IgG1, emGFP expression was found in > 80% of IgG1^+^ class-switched memory B cells and > 70% of plasma cells.

The selective expression of fusion proteins between emGFP and the tetraspanin CD63 in EVs of Cγ1^CD63-emGFP^ reporter mice also enabled tracking and characterization of EVs derived from B cells undergoing transcription at the Cγ1 locus within a normal polyclonal B cell repertoire. CD63 is most commonly utilized for tagging specific EV subtypes with fluorescent reporters^45^. Surface-bound reporters on EVs can be susceptible to enzymatic breakdown, leading to signal loss, alteration of transmembrane protein structure, or may affect EV production due to reporter size^46^. The emGFP reporter was selected for its mutations in the coding regions that prevent aggregation, resulting in a GFP monomer that maintains the signal and minimizes disruption to fusion protein function^17^. CD63-emGFP^loxP/stop/loxP^ reporter mice have effectively enabled cell-type-specific labeling and localization of emGFP^+^ EVs in developing tissues^17^. However, these mice are limited to labeling only those EVs containing the fusion protein. Therefore, in Cγ1^CD63-emGFP^ reporter mice, EVs derived from B cells lacking emGFP will be excluded from the analysis when characterizing EVs based on emGFP expression. Our method for EV isolation utilized differential ultracentrifugation by sedimenting small EVs (< 200 nm) at 118 kG that includes exosomes; thus, our study focused on 118 kG EVs derived from B cells and EVs that may sediment at lesser speeds have been excluded from our studies. CD63 is most widely used to label specific small EV subtypes^46,47^, which may be one reason emGFP^+^ EVs show a smaller size compared to total EVs derived from B cells (Fig. 3a). Furthermore, the differences in size between emGFP^+^ and total EVs may be attributed to the heterogeneity of mature B cell subsets in the spleen that release heterogeneous subtypes of EVs. Future studies exploring the differences between emGFP^+^ and emGFP^-^ EVs hold potential for revealing new insights into the heterogeneity and functional diversity of B cell-derived EVs.

We identified Cγ1^CD63-emGFP^ B cells stimulated with LPS + IL-4 to promote switching to IgG1 induced the release of EVs that expressed IgG, emGFP, and the tetraspanins CD9, CD81, and CD63, suggesting the presence of the fusion protein within EVs. EVs released from Cγ1^CD63-emGFP^ B cells stimulated with LPS alone also expressed IgG, CD9, and CD81. In contrast, low levels of emGFP and CD63 were detected, suggesting inefficient Cre-mediated expression of the fusion protein due to LPS stimulation of murine B cells strongly inducing switching to IgG3 and IgG2b and not IgG1^15,24,25^. Interestingly, reduced expression of CD63 in EVs derived from B cells stimulated with LPS only suggests these EVs are also not enriched in endogenous CD63. CD63 and other tetraspanins have been shown to regulate the mechanisms involved in EV biogenesis and recruitment of selected proteins^45^. These findings raise the possibility that the type of activating signal that a B cell receives influences the developmental pathway used during the formation of EVs. Given that the immunoblot and ELISA methods used in our analyses detect the expression of CD63 and other tetraspanins on a bulk population of emGFP^+^ EVs, the distribution of expression of each tetraspanin across individual EVs isn’t known. Thus, it is possible that emGFP^+^ EVs comprise subtypes that express all or various combinations of tetraspanins.

We also identified that emGFP^+^ EVs expressed surface IgG1, as visualized by ImageStream flow cytometry and measured by ELISA. Although we cannot completely rule out the binding of soluble IgG to Fc receptors present on EVs, several observations suggest that this possibility is unlikely to substantially contribute to surface IgG expression on EVs. First, mature B cells do not express the high-affinity FcγRI (CD64) or low-affinity FcγRII (CD32) and FcγRIII (CD16)^28,29^. This is consistent with our results demonstrating that the majority of emGFP^+^ EVs do not express these Fc receptors and only a small fraction of EVs expressing CD16/32 and/or CD64 were detected (~ 1.6% of the total EV numbers in Fig. 3e). Second, we were unable to detect exogenous monomeric IgG binding to emGFP^+^ EVs. These data suggest that surface IgG on EVs is not likely due to monomeric IgG antibodies binding to CD16/32 and CD64. It is possible that these EVs, like mature B cells, express the inhibitory Fc receptor FcγRIIb (CD32B) that could bind soluble IgG complexes. Whereas FcγRIIb has low binding affinities for monomeric IgG, the affinities for immune complexes are significantly higher^48^. Based on the differences in structure, specificity, and affinity for IgG binding, the potential role of FcγRIIb on B cell-derived EVs to bind soluble IgG requires further investigation. Further, we identified emGFP^+^ EVs in vivo, and our studies indicated that these emGFP^+^ EVs expressed surface IgG that binds specific antigen and additionally binds low molar ratios of antigen suggesting these EVs were released from affinity mature B cells. We also revealed that emGFP^+^ EVs expressing surface IgG that bind the influenza HA antigen can neutralize influenza virus infection in vivo. Lastly, using Cγ1^CD63-emGFP^ reporter lupus-prone mice, we identified GC B cells, switched memory B cells, and plasma cells that express emGFP, which are all B cell subsets that likely contribute to the production of systemic emGFP^+^ EVs with surface IgG autoantibodies to nuclear antigens.

Although our studies and other published studies^8,21^ have both shown that B cell activation is associated with the release of EVs that express surface Ig, our results directly identify IgG1-switched GC B cells and their progeny in vivo are the main source of EVs that express surface IgG1. These differences are likely due to the in vitro methods used for analyzing B cell-derived EVs, as well as many of the experiments in previous studies that used B cell lines^6,7,9,21,49^. While we identified emGFP^+^ GC B cells, switched memory B cells, and plasma cells in secondary lymphoid organs two weeks after immunization with NP-KLH, we cannot rule out that extrafollicular B cell responses also undergo Cre-mediated recombination at earlier time points that may contribute to emGFP^+^ EVs detected in circulation. However, by confocal microscopy, we did not see detectable emGFP^+^ B cells at extrafollicular sites that would suggest recruitment of B cells undergoing transcription at the Cγ1 locus. Further, high affinity binding to NP by emGFP^+^ EVs isolated from sera of immunized Cγ1^CD63-emGFP^ reporter mice suggests an antigen-driven selection of higher-affinity B cells in GCs that differentiate into plasma cells and SWM B cells and release EVs. Future experiments devoted to assessing the extrafollicular B cell response at earlier time points and by increasing the period after immunization when GCs wane will clarify this point.

Our study is the first to our knowledge to directly identify B cell-derived HA-specific IgG^+^ EVs that can neutralize PR8 virus infection in vivo and is consistent with previous reports indicating that antibodies specific for HA neutralize infection in vivo and associate with protection against viral infection in humans^50,51^. Broadly neutralizing antibodies against influenza viruses have been thought to provide protection through their Fab variable region by blocking HA attachment to sialic acids on the surface of host cells or by inhibiting the viral fusion process^52^. A role for the Fc domain of HA-specific IgG to interact with FcγR has also been implicated for protection against influenza virus in vivo^53^. Our results show that HA-specific IgG^+^ EVs isolated from sera contained a minimal amount of soluble IgG supporting the mechanism of viral neutralization involves Fab recognition of HA. However, we do not know whether soluble IgG antibodies specific for HA can also interact with HA bound to IgG on the surface of EVs that may form immune complexes and contribute to protection in vivo by FcγR-mediated cytotoxicity and clearance of infected cells. The binding of IgG^+^ EV immune complexes to various FcγRs such as CD64 and CD16/32, mainly expressed on monocytes/macrophages, dendritic cells, and activated neutrophils, could mediate antibody-dependent cellular phagocytosis and antibody dependent-cellular cytotoxicity. We speculate that full protection and lack of body weight loss observed in mice infected with a high dose of HA-specific EV and PR8 virus mixture is mediated independent of FcγRs, as has been shown for high doses of broadly neutralizing HA-specific mAbs in vivo^53^. Whether HA-specific IgG^+^ EVs neutralize in vivo independently of Fc-FcγR interactions or involve FcγRs for in vivo protection must be further examined.

Analysis of Cγ1^CD63-emGFP^ *Nba2* lupus-prone reporter mice revealed that spontaneous GC B cell responses and elevated serum levels of antinuclear IgG autoantibodies correlated with increased emGFP expression in GC B cells and circulating levels of emGFP^+^ EVs that expressed IgG against nuclear antigens. These results demonstrate that in a separate disease model EVs derived from B cells undergoing IgG1 class switching can express surface IgG1 and recognize specific antigen. Subsets of EVs in plasma of humans with SLE co-express IgG and numerous autoantigens on their surface that are implicated in autoimmunity^54–57^. Similarly, subpopulations of EVs in the serum of MRL-*lpr/lpr* and NZB/W lupus mice express IgG^58^. However, less is known about the cellular source of these EV-associated autoantigens and the nature of IgG expression due to the vast heterogeneity of circulating EVs. Our findings indicate that at least a subset of EVs in serum from lupus-prone reporter mice are derived from spontaneously activated B cells, express surface IgG, and bind nuclear antigens. Future studies with the Cγ1^CD63-emGFP^ *Nba2* reporter mice may improve our understanding of whether IgG^+^ EVs derived from autoreactive B cells contribute to disease by forming immune complexes or by carrying encapsulated cargo molecules to target cells that might lead to inflammation and tissue damage.

Antibody production is achieved through the participation of diverse B cell populations. While this study focused on the production of EVs derived from B cells undergoing IgG1 switching and CD63 expression, we acknowledge that antibodies of other isotypes are also produced. Further study is needed to evaluate the production and nature of EVs that express immunoglobulin isotypes beyond IgG, their B cell origin, and their potential immune function. Recent work provides evidence that peritoneal B1a and B2 cells release EVs that contain IgM after LPS stimulation^11^, supporting the notion that immunoglobulin-containing EVs can be produced by heterogeneous B cell populations. Integrating the emGFP reporter mice with additional B cell Cre-driver mice could be used to address these important questions. In sum, the Cγ1^CD63-emGFP^ reporter mice enable in vivo identification and tracking of activated B cells producing EVs and the EVs they release, which can be used to interrogate the biological features and function of B cell-specific EVs in health and disease states.

## Materials and methods

### Mice

Cγ1^Cre^, CD63-emGFP^loxP/stop/loxP^, and B6.*Nba2* mice have been described^15,17,39,40,42,59,60^. CD63-emGFP^loxP/stop/loxP^ mice and Cγ1^Cre^ mice were first backcrossed to either wild-type C57BL/6 or B6.*Nba2* congenic mice and then intercrossed to generate Cγ1^CD63-emGFP^ B6 mice, Cγ1^CD63-emGFP^ *Nba2* mice, and littermate controls. Mice were all on a C57BL/6 background and those bred with the congenic *Nba2* strain were analyzed by speed congenic genotyping by DartMouse™ to confirm inheritance of the *Nba2* locus on distal Chromosome 1. Experiments used 2- to 7-mo-old, age- and sex-matched mice with a mean weight of 18–22 gm, generated from breeding pairs, were randomly allocated to each experimental or control group. Each experiment was repeated at least three times using separate litters of 5–9 mice. No mice were excluded from analysis. All mice were bred and maintained in the specific-pathogen free animal facilities at the University of Virginia and euthanized by CO_2_ inhalation, followed by cervical dislocation, with the approval of the Institutional Animal Care and Use Committee protocol #3506 and were used in compliance with the Association for Assessment and Accreditation of Laboratory Animals Care policies. All research was conducted in accord with the ARRIVE guidelines.

### In vitro stimulation of B cells

B cells from spleens of mice were purified to greater than 90% using the B Cell Isolation Kit (Miltenyi Biotec, Auburn, CA) according to the manufacturer’s instructions. B cells at 3 × 10^6^ cells/well were cultured in 6-well plates for 3 or 7 days in complete RPMI medium supplemented with 10% heat-inactivated exosome-depleted fetal bovine serum (Biowest, Bradenton, FL), β-mercaptoethanol and antibiotics. Cells were cultured with 10 ng/mL murine IL-4 (ThermoFisher Scientific) alone or in the presence of 10 μg/mL agonistic anti-mouse CD40 mAb (clone FGK4.5; Bio X Cell, Lebanon, NH) or 3 μg/mL LPS (*Escherichia coli* O111; Sigma-Aldrich, St. Louis, MO). Cells were cultured at 37 °C and 5% CO_2_. EVs were isolated from cultured media for further study and emGFP^+^ B cells were analyzed by flow cytometry.

### Immunization of mice

Cγ1^CD63-emGFP^ B6 mice were immunized intraperitoneally with 50 μg NP-KLH (NP32; Biosearch Technologies, Petaluma, CA) in alum on day 0 and day 7, and serum was collected on day 14 as described^61^. Cγ1^CD63-emGFP^ B6 mice were subcutaneously immunized with influenza A virus PR8/34 recombinant HA protein (10 μg/mouse, Sino Biological, Wayne, PA) in the adjuvant Addavax on days 0, 14, and 21. On day 35, mice were euthanized and serum from 6 to 12 immunized mice was pooled and EVs were isolated.

### Influenza viral infection

EVs isolated from sera of Cγ1^CD63-emGFP^ B6 mice immunized with either NP-KLH or recombinant Influenza A/PR8 HA protein (Sino Biological) by differential centrifugation were quantified by nanoparticle tracking analysis and at a high (3 × 10^12^) and low (3 × 10^9^) concentration of particles in a 20 μl volume of PBS were incubated with 20 μl of influenza PR8 virus (~ 400 plaque-forming units per mouse) at 37 °C for 60 min. EV and virus mixtures, or only virus, were infected into 8- to 10-week-old C57BL/6 mice by intranasal injection as previously reported^35^. Infected mice were monitored daily for two weeks for body weight changes and survival rates. Both real death and humane euthanasia when 25% of body weight loss was observed were counted as mouse mortality.

### Flow cytometry

Single-cell suspensions from spleen were depleted of RBCs by ammonium chloride-Tris lysis. Cells (2 × 10^6^) were washed in PBS containing 5% bovine calf serum and incubated with normal rat serum or rat anti-mouse CD16/32 antibody (BD Biosciences, San Diego, CA) diluted to 5% (v/v) to reduce non-specific binding of fluorescent-labeled antibodies. Cells were then stained with the following rat anti-mouse antibodies and reagents: anti-B220-APC-Fire750 (RA3-6B2; BD Biosciences), anti-CD138-APC (281.2; BD Biosciences), anti-CD69-PerCP-Cy5.5 (H1.2F3; eBioscience), anti-CD95-PE (Jo2; BD Biosciences), anti-GL7-EF450 (GL7; Biolegend), anti-IgG1-PE-Dazzle594 (M1-14D12; eBioscience), anti-CD38-EF450 (90; Invitrogen), anti-CD4-APC (GK1.5; Invitrogen), anti-CD3-PE-Cy7 (145-2C11; BD Biosciences), biotin-labeled PNA (Vector Labs, Burlingame, CA), and streptavidin-PE-Cy7 or -PerCP-Cy5.5 (BD Biosciences). Viability was determined using LIVE/DEAD Fixable Aqua or Yellow (ThermoFisher Scientific, Waltham, MA). Stained cells were acquired on an Attune Nxt flow cytometer (ThermoFisher Scientific), with a minimum of 100,000 events collected. Spectral overlap compensation and data analysis were performed using FlowJo software version 10 (Tree Star, Ashland, OR). Profiles are presented as biexponential pseudocolor density plots. Gates were set based on live cells, singlets, and fluorescence-minus-one controls.

### Enzyme-linked immunosorbent assays (ELISA)

Total IgG1 and NP hapten-specific IgG antibodies were measured by ELISA as previously described^62^. Serum from hyperimmunized NP-specific B cell transgenic mice^63^ bred onto the C57BL/6 background was used as a standard in ELISAs to determine the concentration of NP-specific IgG in serum and EV samples^61,64^. Autoantibody IgG antibodies to chromatin, total histones, ssDNA, and dsDNA were measured by ELISA as previously described^65^. Animals were considered positive if they reached levels higher than the mean ± 2 SD of age-matched control B6 mice. For detection of EV markers, plates coated with NP or nuclear antigens were incubated with EVs followed by antibodies against CD9 (KMC8; BD Biosciences), CD63 (NVG-2; BD Biosciences), and CD81 (Eat2; BD Biosciences). These antibodies were detected with biotin-labeled anti-rat or anti-hamster IgG (Jackson ImmunoResearch Labs, West Grove, PA) and streptavidin-HRP (Southern Biotech, Birmingham, AL). GFP was detected with an unlabeled rabbit anti-GFP mAb (coating antibody A-6455; Invitrogen) and a biotin-labeled goat anti-GFP antibody (600–106-215; ThermoFisher), followed by streptavidin-HRP. Influenza A H1N1 (A/PR8/1934) HA protein (11,684-V08H; Sino Biological) was used to detect HA-specific antibodies and EVs. All samples were tested in duplicates at concentrations of dilutional linearity from 1:1000, 1:10,000, and 1:30,000 for serum and 3.3 × 10^7^, 1.1 × 10^7^, and 3.7 × 10^6^ particles/ml for EV samples. Plates were read at 450 nm using a BioTek Plate Reader.

### Immunofluorescence microscopy

For immunofluorescence staining, tissue was snap-frozen, embedded in OCT, and cut into 5 μm sections. Slides were fixed in a 1:1 solution of ethanol:acetone, blocked with PBS containing 3% BSA, and incubated with the following reagents: anti-IgD-PE (11-16c; eBioscience), anti-GL7-PerCP-Cy5.5 (GL7; Biolegend), anti-Thy1.2-APC (53-2.1; eBioscience), anti-B220-PerCP-Cy5.5 (RA3-6B2; Biolegend), and Hoechst for labeling nuclei. Sections were analyzed using a Zeiss LSM 900 Confocal microscope and the National Institutes of Health ImageJ and Zeiss software. Scale bars are provided in each image.

### EV isolation

Methods of EV isolation and characterization were performed using the guidelines established by the International Society for Extracellular Vesicles^66^. EVs were isolated by sequential centrifugation steps at different centrifugal forces adapted from the study by Raposo et al. for isolating EVs from culture medium of B cell lines using optimized rotor type, *g*-force and centrifugation times^9,67^. Using this approach, EVs from B cells separated by their sedimentation at 118,000 × *g* were analyzed in our studies (118 kG EVs). Blood was collected from mice via retroorbital puncture and after coagulation spun at 13,000 × *g* for 5 min at room temperature to isolate serum. The serum was transferred to a clean 1.5 mL microcentrifuge tube and spun again at 13,000 × *g* for 10 min to ensure no cell or platelet contamination. Serum samples from groups of mice were pooled and spun at 10,000 × *g* for 45 min at 4 °C. The remaining cell-free serum was resuspended in 10 mL of PBS, transferred to Beckman Coulter thin wall, 12 mL polypropylene centrifuge tubes, and spun at 118,000x*g* for 70 min at 4 °C using a Beckman Coulter Optima LE-80 K Ultracentrifuge with SW-41Ti rotor (k-factor: 217.5). For isolating EVs derived from B cells in vitro, splenic B cells were grown as described above. Culture media was spun at 250 × *g* for 5 min to remove cells. The supernatant was transferred to clean 15 mL polystyrene tubes and spun at 2000 × *g* for 15 min at 4 °C to remove dead cells. The supernatant was transferred to new tubes and spun at 10,000 × *g* for 30 min at 4 °C. The supernatant was transferred to polypropylene centrifuge tubes and ultracentrifuged at 118,000 × *g* for 70 min at 4 °C. The EV pellet was resuspended in 50 μL PBS and stored at − 80 °C no more than 3 months to maintain ~ 94% EV recovery (Supplementary Fig. S12). Thawed EVs were subjected to nanoparticle tracking analysis and analyzed by ELISA, western blot, or size exclusion chromatography using 70 nm Gen2 qEV Sepharose columns (Izon, Medford, MA), following the manufacturer’s protocol. The qEV columns were equilibrated with sterile PBS and 0.5 ml fractions were collected. Aliquots from each fraction were tested by nanoparticle tracking analysis or ELISA to determine particle number and total IgG concentrations.

### EV quantification

Throughout the entire study, the size and concentration of EVs isolated from media and sera was determined by nanoparticle tracking analysis using a ZetaView PMX 120 equipped with a 488 nm laser and a long wave pass filter with a 500 nm cutoff and CMOS camera (Particle Metrix, Ammersee, Germany). Each sample was diluted in 10 mM HEPES, 2.5 mM EDTA buffer (pH 7.0) to an appropriate concentration before being analyzed. Duplicates of each sample were measured at 11 different positions, three times. Samples were recorded at 25 °C with a shutter speed of 30 frames per second and a camera sensitivity of 75. EVs were measured both in bright field (total) and using a 488 nm filter (GFP^+^) alongside buffer only controls to ensure no EV contamination from the buffer. The counts and size of particles were automatically calculated by the software.

### Electron microscopy of EVs

EVs isolated from cell culture medium of stimulated B cells were analyzed by cryo-electron microscopy for direct visualization of vesicles. The EV sample (~ 3.0 μL) was applied to glow-discharged lacey carbon grids and vitrified using a Vitrobot Mark IV (ThermoFisher Scientific) with back side blotting. Grids were imaged at the University of Virginia Molecular Electron Microscopy Core using a 200 kV Glacios, equipped with an XFEG electron source and Falcon 4 direct electron detector.

### Western blot analysis

EV and cultured B cell samples were lysed with RIPA buffer (Millipore, Burlington, MA) in the presence of protease inhibitors (P8340, Sigma Aldrich) and the protein concentration was determined using the Pierce BCA Protein Assay Kit (ThermoFisher Scientific) according to the manufacturer’s instructions. Equivalent amounts of lysate (5–10 μg) were prepared in non-reducing Laemmli SDS sample buffer or reducing Laemmli SDS sample buffer with 5% 2-mercaptoethanol (ThermoFisher Scientific)^68^, heated for 10 min at 100 °C, and loaded on 4–20% or 4–12% polyacrylamide gels (Fisher Scientific). Protein gels were transferred onto nitrocellulose membranes, blocked with 5% nonfat dried milk for 1.5 h, and incubated with primary antibody diluted in 5% nonfat dried milk in TBST overnight at 4 °C on a rocker using the following antibodies specific for: CD9 (1:1000; 98327S, Cell Signaling Technology), CD81 (1:500; 10037S, Cell Signaling Technology), CD63 (1:1000; ab217345, Abcam, Boston, MA), EGFP (4 μg /mL; OSE00003G, ThermoFisher), IgG H&L chains (1:2000; ab6709, Abcam). Membranes were washed 3 × in TBST and incubated with secondary detection goat anti-rabbit IgG HRP antibody (1:10,000; ab97051, Abcam or 1:5000; 111-035-144, Jackson Immunoresearch Laboratories) diluted in 5% nonfat dried milk in TBST overnight at 4 °C on a rocker. Blots were scanned using a BioRad ChemiDoc MP Imaging System. Primary ab6709 and secondary ab97051 IgG antibodies were confirmed to not have detectable non-specific binding (Supplementary Fig. S6c). Protein loading was confirmed by Ponceau S staining (Cell Signaling Technology, Danvers, MA) according to manufacturer’s instructions.

### ImageStream X analysis of EVs

Methods for analyzing EVs by ImageStream were performed using the MIFlowCyt-EV guidelines for standardized reporting of EV flow cytometry experiments^69^. Purified EV samples were spun at 12,000x*g* for 5 min at room temperature in 0.22 μm Ultrafree-MC centrifugal filter units (EMD Millipore) and stained in 100 μL of 0.1 μm filtered PBS with 0.125 μg anti-IgG1-Alexa Fluor 647 (RMG1-1; Biolegend) or anti-CD16/32-Alexa Fluor 647 (93; Biolegend), 0.125 μg anti-CD64-PE (X54-5; Biolegend), and 0.125 μg mouse IgG-DyLight 405 (ThermoFisher Scientific) for 2 h at room temperature. All antibodies were spun at 17,000x*g* for 10 min to remove aggregates before staining. Stained samples were serial diluted fivefold in 0.1 μm filtered PBS to avoid swarm detection to achieve 1 event per frame and acquired for 5 min on an ImageStream X Mark II (Cytek Amnis) at a 60 × magnification with a 7 μm core size and low flow rate, according to methods previously established^70–72^. emGFP fluorescence was excited with a 488 nm laser at 200 mW and emission was collected using a 480–560 nm bandpass filter, PE fluorescence was collected using a 595–650 nm bandpass filter excited with a 561 nm laser at 200 mW, and AF647 fluorescence was collected using a 660–745 nm bandpass filter excited with a 642 nm laser at 150 mW. Brightfield was collected using a 430–480 nm bandpass filter and SSC/Darkfield detection was collected using a 745–800 nm bandpass filter excited with a 785 nm laser at 70 mW. Antibodies alone in buffer and unstained EVs were run as controls as previously described^73^. Buffer only was run prior to each sample for the same acquisition time to measure background and to ensure there was no contamination between samples. Cells in peritoneal washes with PBS from wild-type C57BL/6 mice (2 × 10^6^) were stained in 100 μL PBS containing 1% bovine serum albumin (BSA) and incubated for 10 min at room temperature in the absence or presence of 5 μg rat anti-mouse CD16/32 antibody (2.4G2; BD Biosciences, San Diego, CA) to block mouse IgG Fc binding to Fc gamma receptors. Cells were then stained in 100 μL 1% BSA PBS with 0.125 μg mouse IgG-DyLight 405 and either 0.125 μg rat anti-mouse B220-FITC (RA3-6B2; BD Biosciences) or 0.125 μg rat anti-mouse CD11b-FITC (M1/70; BD Biosciences) for 30 min at room temperature. Cells were then washed with 1% BSA PBS and acquired on an ImageStream X Mark II at a 60 × magnification using the same laser settings as described for EV acquisition. Data analysis was performed using IDEAS software v6.1.

### Statistical analyses

Statistics were determined using Prism software v10 (GraphPad Software, Boston, MA). To assess differences between groups, the unpaired, two-tailed Students *t-*test was used. Differences in survival between groups were calculated by log-rank test. Data shown are mean ± SEM. Significance was defined as *p* values ≤ 0.05 and are stated in the figure legends.

### Supplementary Information

Supplementary Figures.

## Data Availability

All data supporting the findings of this study are available within the paper and its Supplementary Information.

## Abbreviations

BCR: B cell receptor
Cγ1: Immunoglobulin heavy constant gamma 1
emGFP: Emerald green fluorescence protein
EV: Extracellular vesicles
GC: Germinal center
HA: Hemagglutinin
IgG: Immunoglobulin G
IL-4: Interleukin 4
LPS: Lipopolysaccharide
*Nba2*: New Zealand black autoimmune locus 2
NP-KLH: 4-hydroxy-3-nitrophenylacetyl-keyhole limpet hemocyanin
PNA: Peanut agglutinin
PC: Plasma cell
SWM: Switched memory
