# Supplementary material for: B cells secrete functional antigen-specific IgG antibodies on extracellular vesicles
Source: Sci Rep. 2024 Jul 23;14:16970. doi: 10.1038/s41598-024-67912-y (PMC11266516; doi:10.1038/s41598-024-67912-y)
Supplement: Supplementary file 1 — Supplementary Figures. [file 41598_2024_67912_MOESM1_ESM.pdf]

Supplementary Information

Title: B cells secrete functional antigen-specific IgG antibodies on extracellular vesicles

Authors: Claudia Rival[1,2], Mahua Mandal[1,2], Kayla Cramton[1,2], Hui Qiao[1,2], Mohd Arish[1,3], Jie Sun[1,3], James V. McCann[4], Andrew C. Dudley[2,5], Michael D. Solga[6], Uta Erdbrügger[7], and Loren D. Erickson[1,2,*]

[1]Beirne Carter Center for Immunology Research, University of Virginia, Charlottesville, VA, 22908, USA; [2]Department of Microbiology, Immunology, and Cancer Biology, University of Virginia, Charlottesville, VA, 22908, USA; [3]Division of Infectious Diseases and International Health, Department of Medicine, University of Virginia, Charlottesville, VA, 22908, USA; [4]Department of Cell Biology & Physiology, University of North Carolina at Chapel Hill, Chapel Hill, NC, 27599, USA; [5]Emily Couric Cancer Center, University of Virginia, Charlottesville, VA, 22908, USA; [6]Flow Cytometry Core, University of Virginia, Charlottesville, VA, 22908, USA; [7]Division of Nephrology, Department of Medicine, University of Virginia, Charlottesville, VA, 22908, USA

***Correspondence:**

Loren D. Erickson

PO Box 801386, Charlottesville, VA, 22908, USA

Email: lde9w@virginia.edu

Phone: 434-982-0756

Fax: 434-924-1221

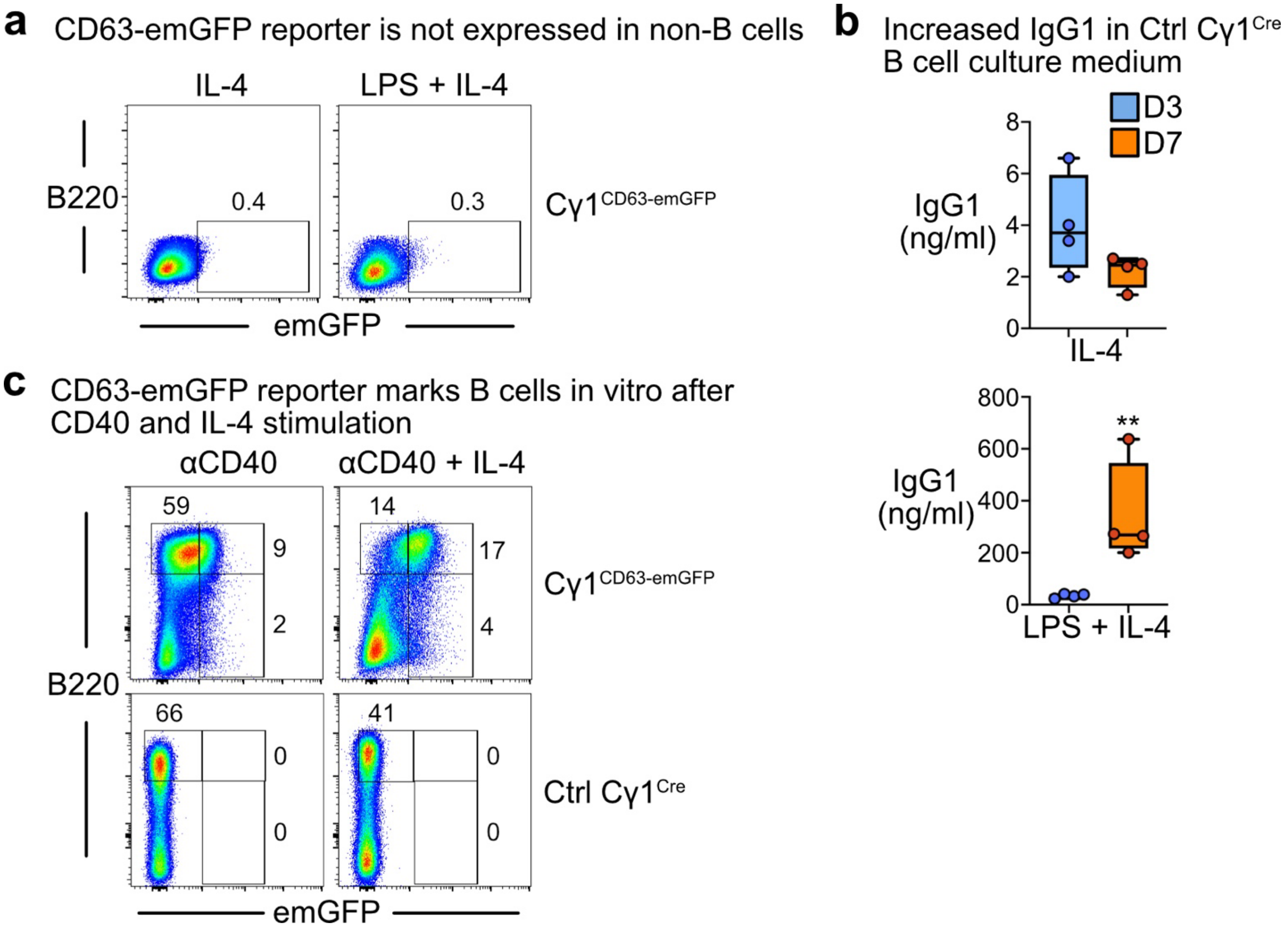

Supplementary Figure S1: Analyzing emGFP expression during in vitro B cell activation.

a, Percentage of non-B cells that express emGFP gated on B220$^-$ cells from Cγ1$^{CD63\text{-}emGFP}$ mice following stimulation with IL-4 or LPS+IL-4 for 3 days. **b**, Concentration of IgG1 in the culture medium of B cells from Cγ1^{Cre} control mice following stimulation with IL-4 or LPS+IL-4 for 3 and 7 days. **c**, Percentage of emGFP$^+$ B cells from Cγ1$^{CD63\text{-}emGFP}$ mice and Cγ1^{Cre} littermate control spleen cells following stimulation with agonistic anti-CD40 mAb or anti-CD40 mAb+IL-4 for 3 days. All data are expressed as mean ± SEM. Results shown are representative of at least three independent experiments. *P* = **-.01, with unpaired, two-tailed *t*-test.

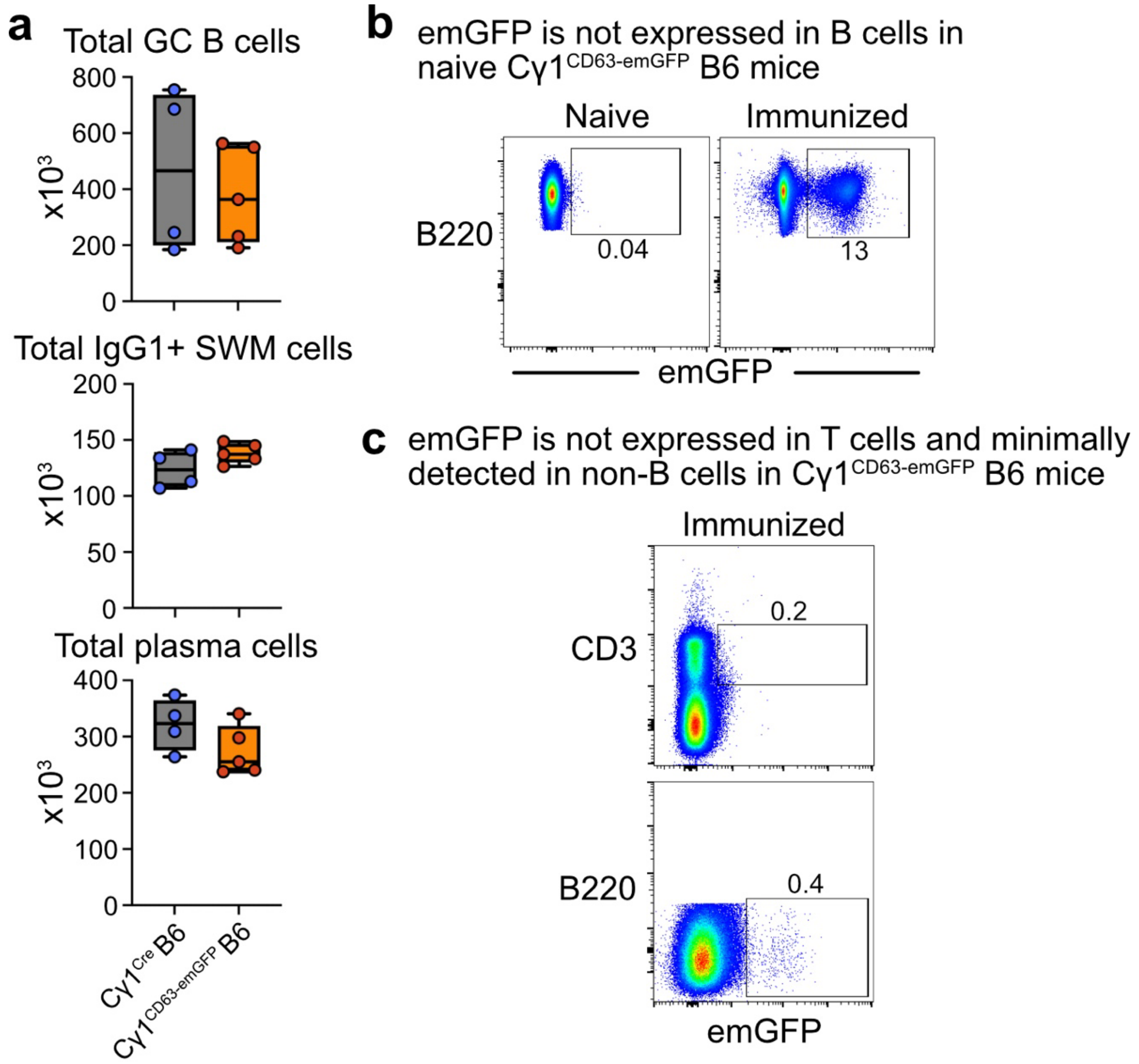

Supplementary Figure S2: emGFP expression in vivo is restricted to B cells in Cγ1$^{\text{CD63-emGFP}}$ mice.

a, Numbers of total GC B cells, IgG1+ switched memory (SWM) B cells, and plasma cells in the spleens of Cγ1$^{\text{CD63-emGFP}}$ and Cγ1^{Cre} control littermates immunized with NP-KLH for 14 days. **b**, Percentage of emGFP^{+} B cells in the spleens of naïve Cγ1$^{\text{CD63-emGFP}}$ mice and after immunization with NP-KLH for 14 days. **c**, Percentage of emGFP^{+} T cells and non-B cells gated on B220^{-} cells in the spleens of Cγ1$^{\text{CD63-emGFP}}$ mice immunized with NP-KLH for 14 days. Results shown are representative of three independent experiments with *n* = 4-5 mice per group.

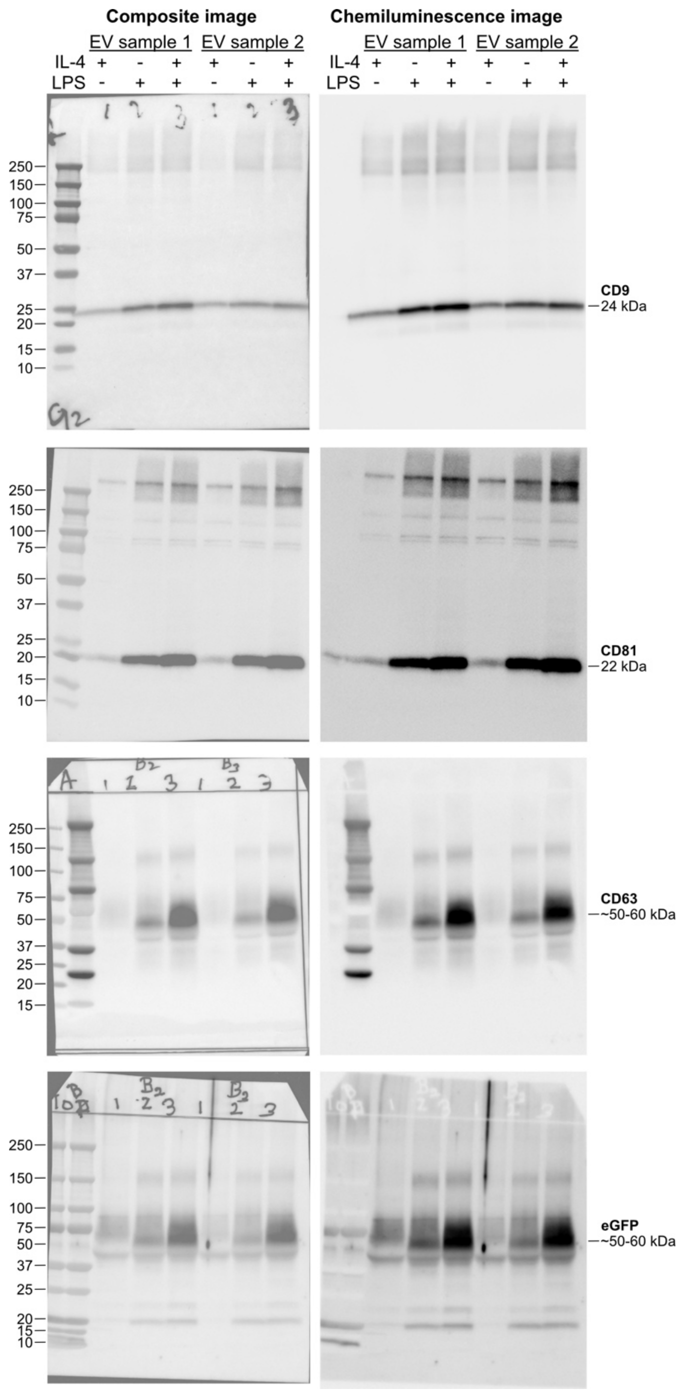

Supplementary Figure S3: Western blots for CD9, CD81, CD63 and emGFP. Lysates from EVs isolated from cell culture medium from Cγ1$^{CD63\text{-}emGFP}$ B cells stimulated with IL-4, LPS, and LPS+IL-4 for 7 days were probed by Western blot for the presence of CD9, CD81, CD63, and eGFP under non-reducing conditions (10 μg/lane). Full composite images with the MW marker in kDa and chemiluminescence images of the indicated protein size are shown for the bands in Figure 3c.

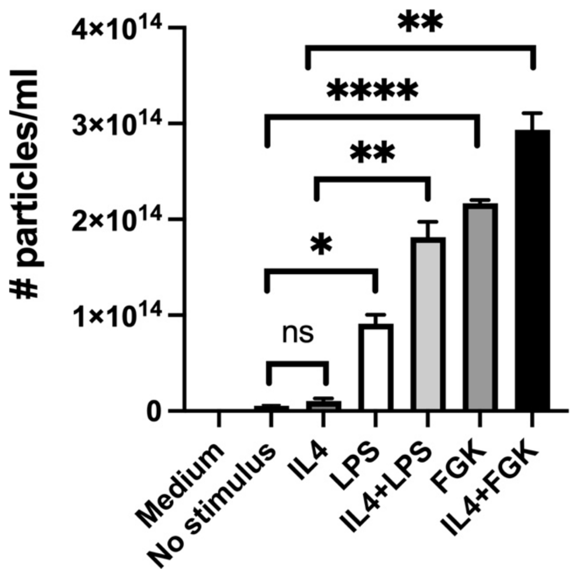

Supplementary Figure S4: EV secretion from B cells is induced with LPS and anti-CD40 mAb stimulation. Nanoparticle tracking analysis of EVs isolated from cell culture medium from $C\gamma 1^{CD63\text{-}emGFP}$ B cells unstimulated or stimulated with IL-4, LPS, LPS+IL-4, anti-CD40 mAb (FGK), and FGK+IL-4 for 7 days. Nanoparticle tracking analysis of culture medium alone served as a background control. All data are expressed as mean ± SEM. Results shown are representative of three independent experiments. *P* = *0.05, **0.01, ****0.0001 with unpaired, two-tailed *t*-test

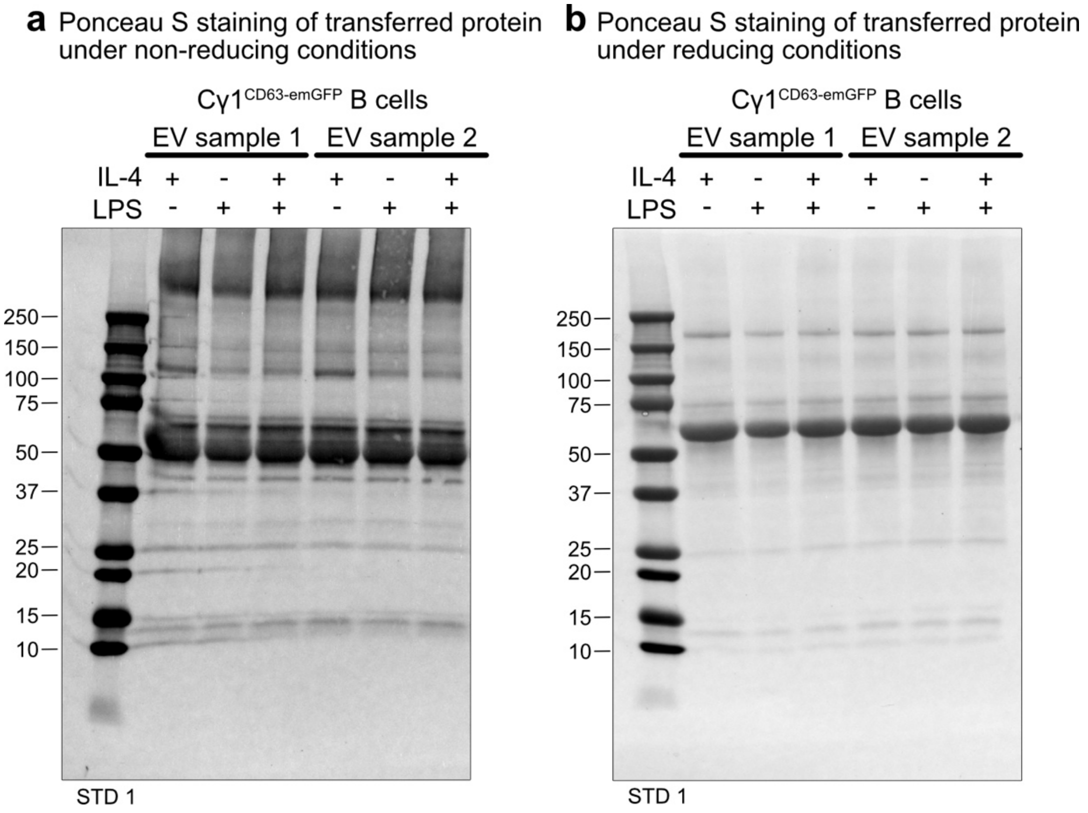

Supplementary Figure S5: EV lysate samples show equivalent protein loading on nitrocellulose membranes. a,b, Lysates from EVs isolated from culture medium of $C\gamma1^{CD63\text{-}emGFP}$ B cells stimulated with IL-4, LPS, or LPS+IL-4 for 7 days show equivalent protein loading under non-reducing (a) and reducing (b) conditions by Ponceau S staining. STD 1 = Bio-Rad Precision Plus Protein standard.

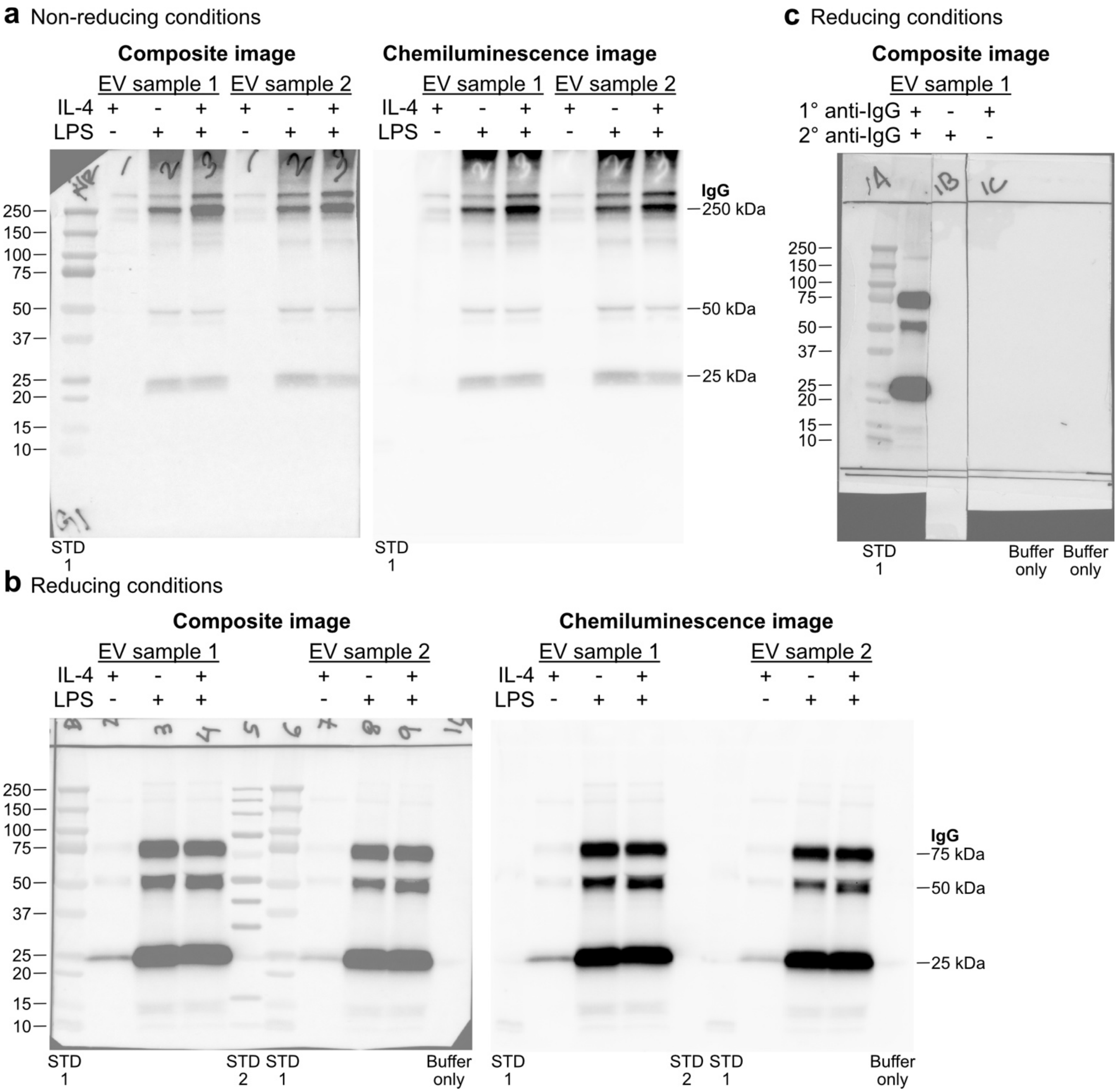

Supplementary Figure S6: Western blots for IgG. Lysates from EVs isolated from cell culture medium from $C\gamma1^{CD63\text{-}emGFP}$ B cells stimulated with IL-4, LPS, and LPS+IL-4 for 7 days were probed by Western blot for the presence of IgG under non-reducing (panel a) and reducing conditions (panel b), loaded equally at 10 μg/lane. Full composite images with MW markers in kDa using STD 1 and chemiluminescence images of IgG protein sizes are shown for the bands in Figure 3d. Panel c shows no non-specific binding of either primary ab6709 or secondary ab97051 antibodies. STD 1 = Bio-Rad Precision Plus Protein standard; STD 2 = Cell Signaling Protein Marker, Broad Range.

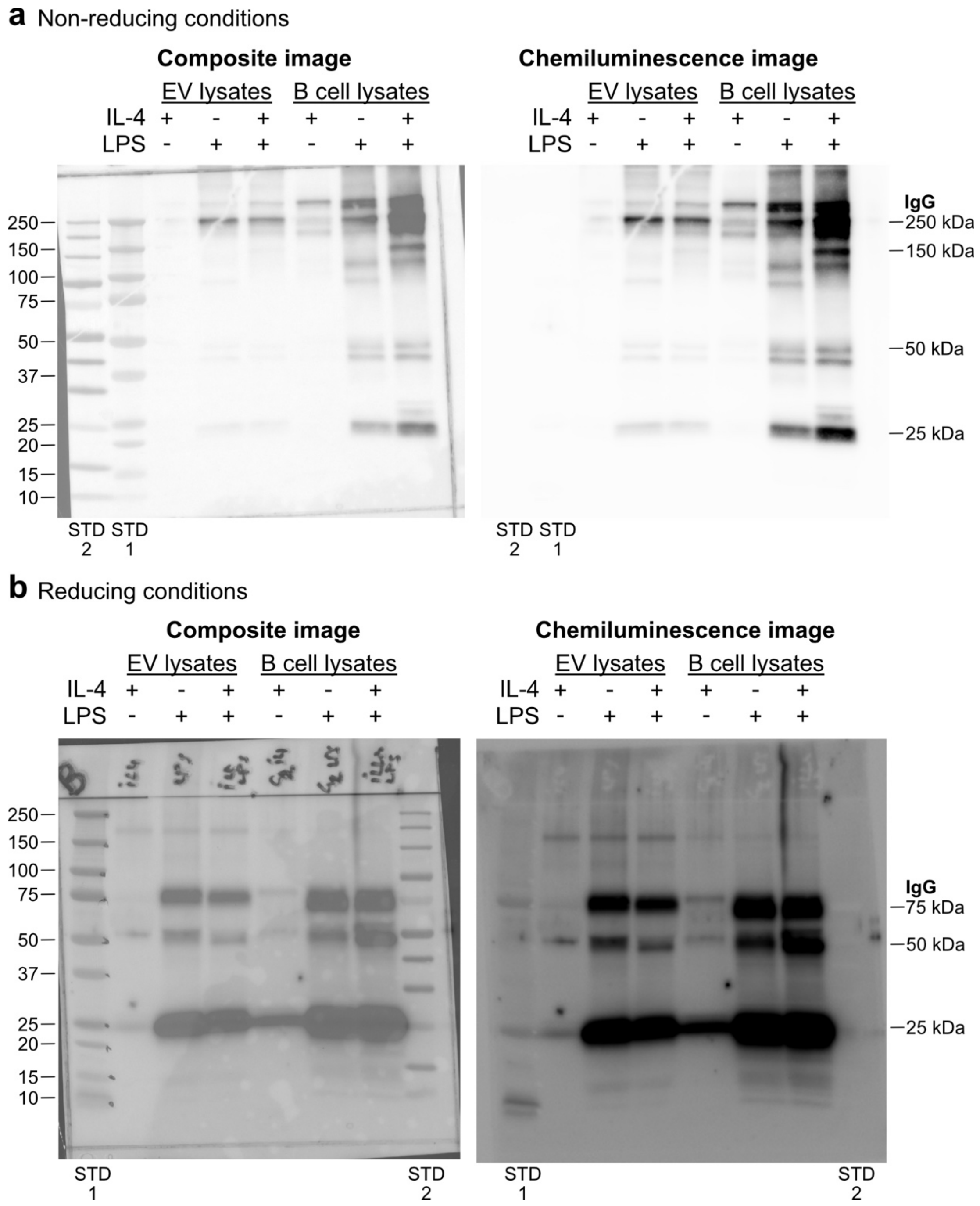

Supplementary Figure S7: Western blots comparing EV and B cell lysates for IgG. Lysates from EVs and B cells isolated from cell culture medium from $C\gamma1^{CD63\text{-}emGFP}$ B cells stimulated with IL-4, LPS, and LPS+IL-4 for 7 days were probed by Western blot for the presence of IgG under non-reducing (panel a) and reducing conditions (panel b), loaded equally at 10 μg/lane. Full composite images with MW markers in kDa using STD 1 and chemiluminescence images of IgG protein sizes are shown. STD 1 = Bio-Rad Precision Plus Protein standard; STD 2 = Cell Signaling Protein Marker, Broad Range.

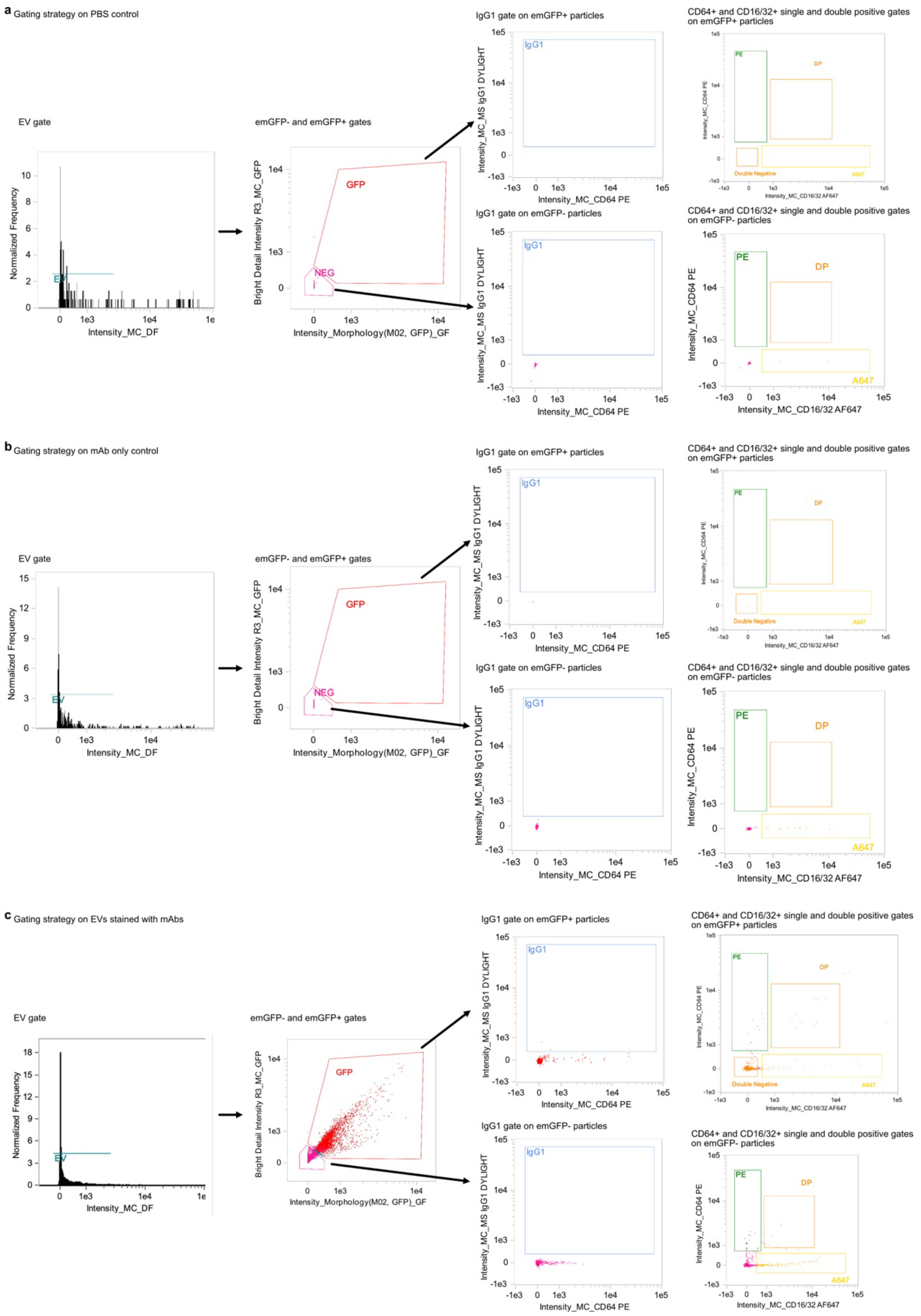

Supplementary Figure S8: Manual gating strategy for analyzing EVs using ImageStream. a-c, EV phenotyping was performed with gates set on emGFP- and emGFP+ EVs based on PBS (a) and mAb stain only (b) controls. **c**, EVs stained with mAbs against IgG1, CD64, CD16/32, and mouse IgG1.

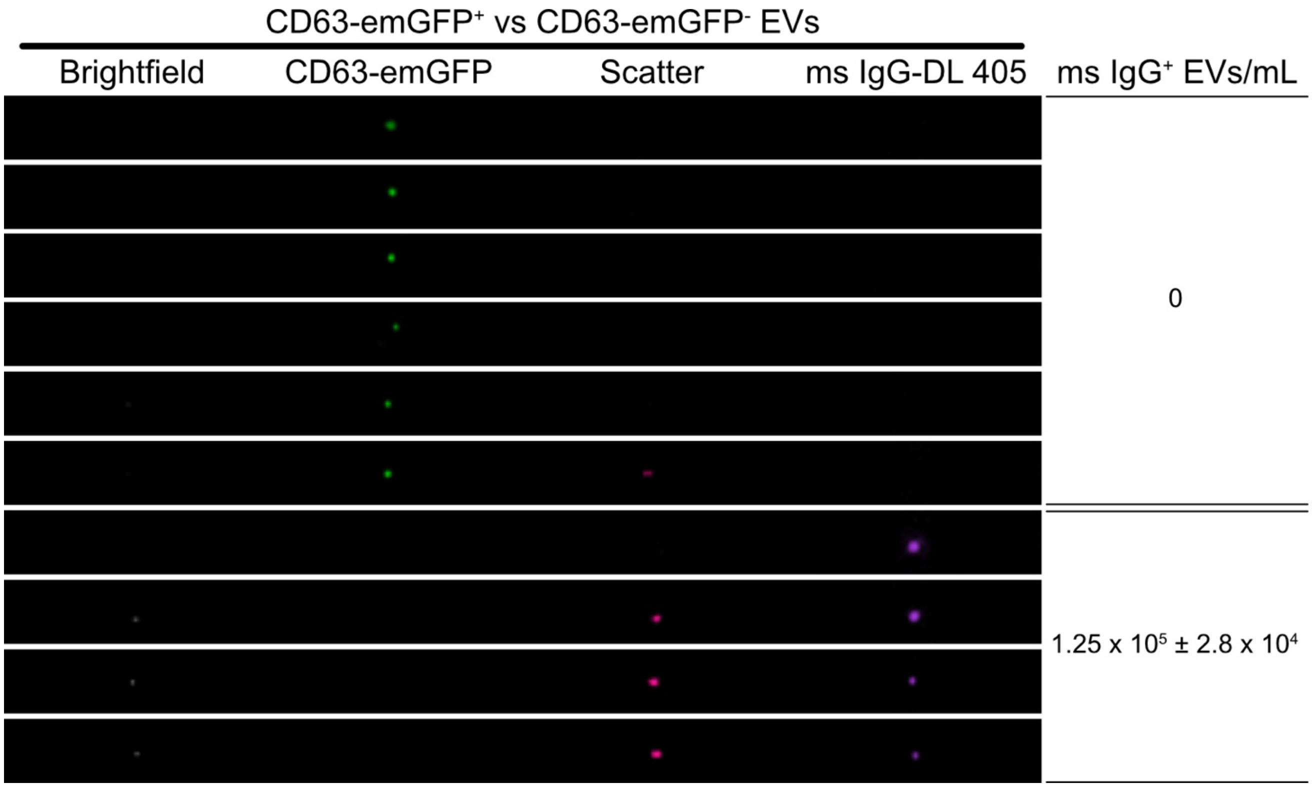

Supplementary Figure S9: emGFP$^-$ EVs but not emGFP$^+$ EVs bind exogenous mouse IgG. ImageStream analysis of individual emGFP$^+$ and emGFP$^-$ EVs isolated from the cell culture medium of Cγ1$^{CD63\text{-}emGFP}$ spleen cells stimulated with LPS+IL-4 for 7 days and stained for binding of exogenous mouse IgG (DyLight 405). emGFP$^+$ EVs ($1.6 \times 10^6 \pm 8.4 \times 10^4$ EVs/mL) do not show detectable mouse IgG binding whereas emGFP$^-$ EVs, detected by brightfield and scatter, in the same sample bind mouse IgG. The results shown used the gating strategy and controls shown in Supplementary Fig. S8 and are representative of three independent experiments.

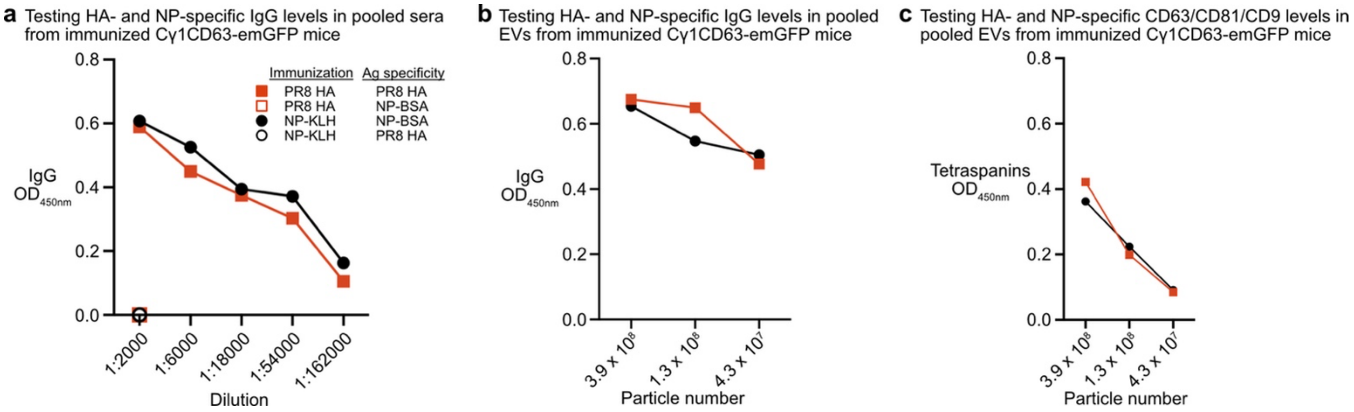

Supplementary Figure S10: EVs isolated from sera from Cγ1$^{CD63\text{-}emGFP}$ mice immunized with PR8 HA or NP-KLH protein show specific antigen binding and tetraspanin expression. a,b, ELISA measurements of NP- and HA-specific IgG antibody levels in whole serum (a) and EVs isolated from sera (b) from Cγ1$^{CD63\text{-}emGFP}$ mice immunized with PR8 HA protein or NP-KLH for 21 days. **c**, ELISA measurements of NP- or HA-specific tetraspanin expression levels in pooled EVs from the same mice.

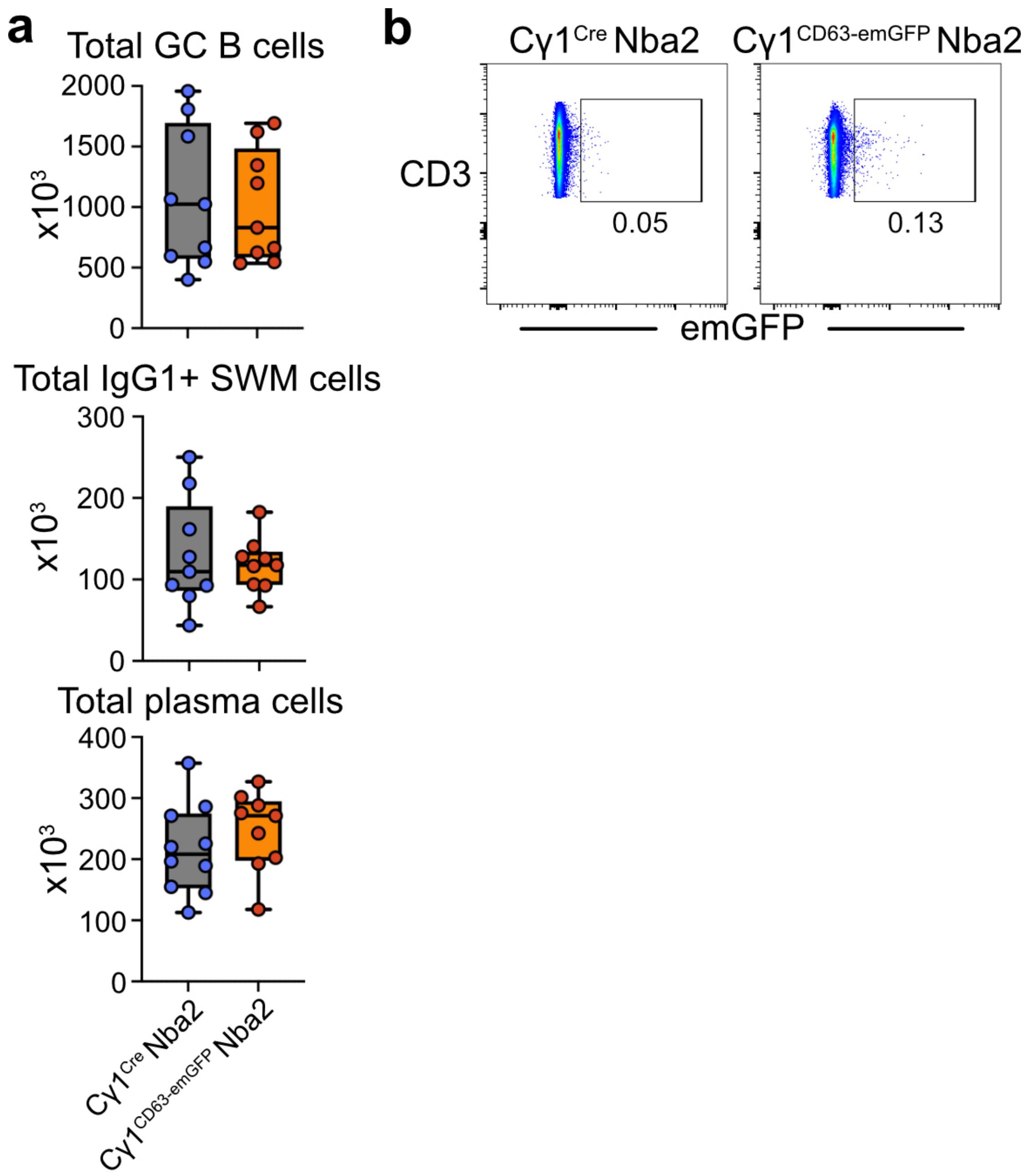

Supplementary Figure S11: Expression of the CD63:emGFP transgene in vivo does not affect spontaneous B cell responses and is not expressed in T cells in Cγ1$^{CD63\text{-}emGFP}$ *Nba2* mice. a, Numbers of total GC B cells, IgG1+ switched memory (SWM) B cells, and plasma cells in the spleens of 7-mo-old Cγ1$^{CD63\text{-}emGFP}$ Nba2 and Cγ1^{Cre} Nba2 control littermates. **b**, Percentage of emGFP^{+} T cells gated on CD3^{+}B220^{-} cells in the spleens of 7-mo-old Cγ1$^{CD63\text{-}emGFP}$ *Nba2* mice and Cγ1^{Cre} *Nba2* littermate controls. Results shown are representative of two independent experiments with *n* = 9 mice per group.

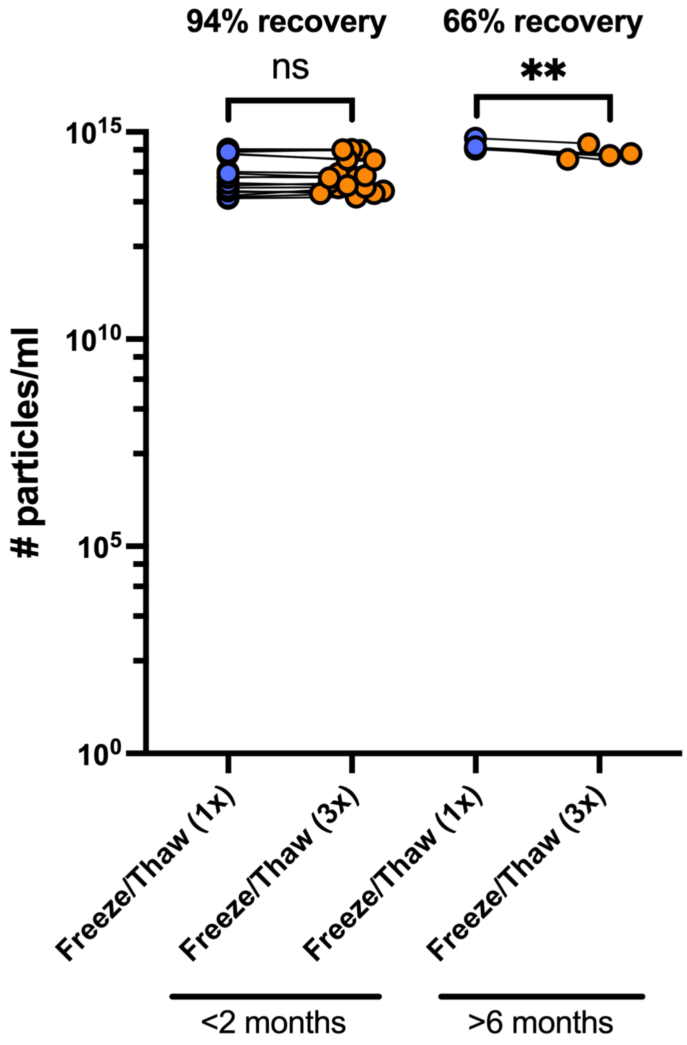

Supplementary Figure S12: EV recovery following freeze/thaw over time. Nanoparticle tracking analysis of EVs isolated from cell culture medium from B cells stimulated with LPS+IL-4 for 3 days and frozen at -80°C and thawed once compared to three times when stored for <2 months or >6 months. *P* = **0.0082, with paired, two-tailed *t*-test. ns = not significant.
